# Design, Synthesis, and Molecular Docking Studies of Novel Pyrazoline-Thiazoles as Cholinesterase Dual-Target Inhibitors for the Treatment of Alzheimer’s Disease

**DOI:** 10.1021/acsomega.5c01055

**Published:** 2025-08-22

**Authors:** Betül Kaya, Ulviye Acar Çevik, Bilge Çiftçi, Adem Necip, Mesut IŞIK, Ebru Nur Ay, Süleyman Yur, Yusuf Özkay, Şükrü Beydemir, Zafer Asım Kaplancıklı

**Affiliations:** † Vocational School of Health Services, Pharmacy Services, 121945 Bilecik Şeyh Edebali University, 11230 Bilecik, Turkey; ‡ Department of Pharmaceutical Chemistry, Faculty of Pharmacy, 52944 Anadolu University, 26470 Eskişehir, Turkey; § Medicinal Plant, Drug and Scientific Research and Application Center (AUBIBAM), Anadolu University, 26470 Eskişehir, Turkey; ∥ Vocational School of Health Services, Bilecik Şeyh Edebali University, 11230 Bilecik, Turkey; ⊥ Vocational School of Health Services, Pharmacy Services, 52966 Harran University, 63300 Şanlıurfa, Turkey; # Department of Bioengineering, Faculty of Engineering, Bilecik Şeyh Edebali University, 11230 Bilecik, Turkey; ¶ Department of Molecular Biology and Genetics, Faculty of Engineering and Natural Sciences, 469683 İstinye University, 34396 İstanbul, Turkey; ∇ Department of Biochemistry, Faculty of Pharmacy, Anadolu University, 26470 Eskişehir, Turkey; ○ The Rectorate of Bilecik Şeyh Edebali University, 11230 Bilecik, Turkey

## Abstract

Ten novel pyrazoline-thiazole derivatives were synthesized and assessed for their potential as acetylcholinesterase and butyrylcholinesterase inhibitors. The structure of the target compounds was characterized by ^1^H NMR and ^13^C NMR, and purity was determined using HPLC. The in vitro enzyme inhibitory activity assays determined that compounds **3f** (IC_50_ = 0.382 μM) and **3g** (IC_50_ = 0.338 μM) showed good inhibitory activity of acetylcholinesterase (AChE). Compound **3f** has a selective inhibitory effect on AChE, while compound **3g** has a dual effect, being effective against both AChE and BChE (IC_50_ = 2.087 μM). The molecular docking results of compound **3g** with high inhibitory activity for AChE experimentally showed that it has a strong inhibitory effect close to that of the reference inhibitor tacrine. The compound **3g** was found to have the highest activity in its interaction with the BChE (4BDS) protein with a low docking score (−5.555 kcal/mol). Furthermore, the prediction of ADME properties of compounds **3f** and **3g** was determined through Swiss ADME.

## Graphical abstract

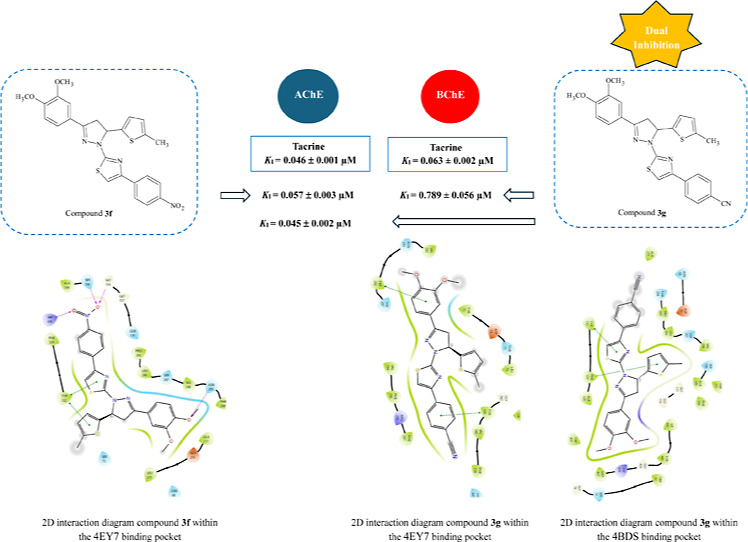

## 1 Introduction

Alzheimer’s disease (AD) is the most commonly diagnosed type of dementia worldwide and is a progressive neurodegenerative disease characterized by cognitive, functional, and behavioral impairments.^1,2^ There are currently approximately 24 million Alzheimer’s patients worldwide. The pathogenesis of the disease has been classified as the amyloid cascade hypothesis,^3,4^ the tau hyperphosphorylation hypothesis,^5^ and the cholinergic hypothesis, with other contributing links.^6–8^ The cholinergic hypothesis is the oldest theory on the pathogenesis of AD.^9^ This hypothesis has made acetylcholinesterase (AChE) and butyrylcholinesterase (BChE) enzymes a therapeutic target for AD. In AD, acetylcholine (ACh), whose levels are significantly reduced due to disruption of the cholinergic metabolic pathway, plays a vital role in cognitive processes. Inhibition of cholinesterases helps alleviate cognitive symptoms by increasing ACh levels in the brains of AD patients.

Current AD therapy strategies primarily depend on the inhibition of AChE to increase ACh levels.^10,11^ However, with the progression of AD, AChE activity decreases and BChE activity increases.^12–14^ Therefore, dual inhibitors that simultaneously inhibit AChE and BChE are of great interest since single-target cholinesterase inhibitors may not provide optimal efficacy. This strategy also serves as a disease-modifying agent, delaying the formation of Aβ plaques.^14–17^ Acetylcholinesterase inhibitors (AChEI) prescribed for the treatment of AD provide only symptomatic treatment. They can also cause gastrointestinal side effects such as diarrhea, vomiting, and nausea, and side effects such as bradycardia, orthostatic hypotension, fatigue, insomnia, anorexia, and weight loss.^3,18,56^ Therefore, the discovery of novel alternative inhibitors is of great importance in the search for more effective and safer treatments for AD.

Nitrogen-containing heterocycles have recently been the focus of attention in medicinal chemistry and drug design research due to their versatile nature and various biological potentials.^19^ Of N-heterocyclic compounds, pyrazolines constitute structural underpinnings of innumerable medications and act as antidepressant,^20^ anticancer,^21,22^ antimicrobial,^22^ antiepileptic,^23^ anti-inflammatory,^24^ anticholinesterase,^25–27^ antioxidant,^28^ etc. The pyrazoline ring is a privileged scaffold for drug design in the management of neurodegenerative disorders due to its ability to interact with pivotal residues of a plethora of crucial biological targets.^29–31^ Particularly, 1,3,5-trisubstituted-2-pyrazolines, embedded with a variety of functional groups have been reported to inhibit molecular targets such as cholinesterases, carbonic anhydrases, and MAOs that are important for the treatment of neurodegenerative disorders such as Parkinson’s disease and Alzheimer’s Disease (AD).^32–37^ 2-Pyrazoline, particularly, is a well-known pharmacophore for designing novel AChE and BChE inhibitors for the treatment of AD. Furthermore, several 3,5-diarylpyrazolines with nanomolar-range ChE inhibitory activity have been reported in the literature.^32,33,36,38–43^

The thiazole ring is also an outstanding scaffold in medicinal chemistry for the development of anti-Alzheimer agents with ChE inhibitory activity.^44–46^ On the basis of these findings, encouraged by previously reported pyrazoline-thiazoles (Figure 1) with promising ChE inhibitory activity,^26,40,41^ a series of the above-mentioned 2-pyrazoline and thiazole hybrid compounds were designed and synthesized in order to identify potential lead candidates as inhibitors of cholinesterase enzymes. To discuss the effect of different substituents on activity, the compounds were designed through substitution with various electron-donating and electron-withdrawing groups from the phenyl ring at the 5-position of the thiazole ring attached to N1 of the 2-pyrazoline ring. In addition, in order to support the experimental results with in silico data, molecular docking calculations were used to evaluate the activities of the synthesized molecules against AChE and BChE proteins. Then, ADME/T calculations were performed to evaluate the effects and reactions of these molecules in the context of human metabolism.

**Figure 1:**
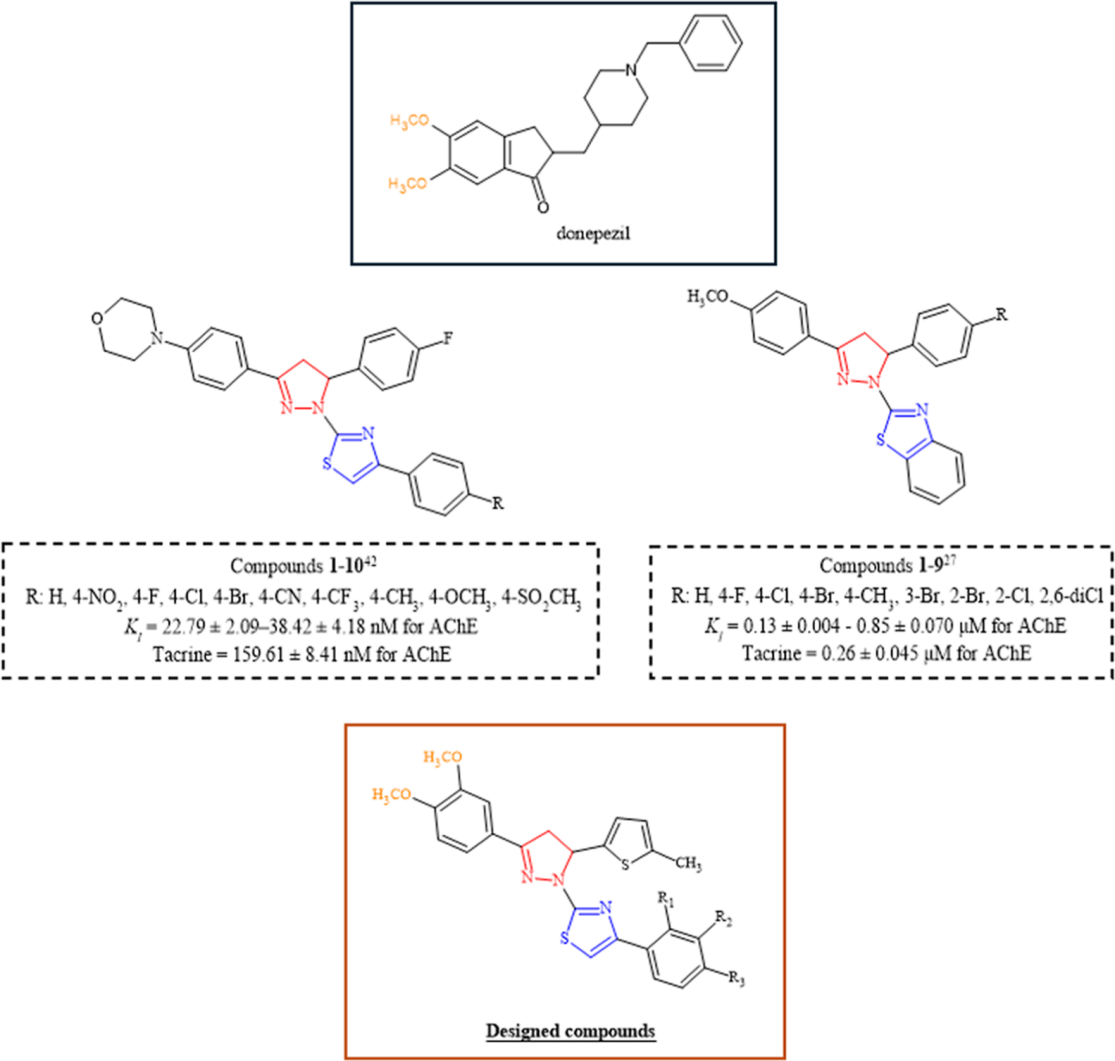
Structures of donepezil, some previously reported AChE inhibitors, and the designed compounds.

## 2 Results and Discussion

### 2.1 Chemistry

The stepwise synthesis of pyrazoline-thiazoles (**3a**–**3j**) derivatives is expressed in Scheme 1. In the first step, 5-methylthiophene-2-carbaldehyde and 3,4′-dimethoxyacetophenone were reacted using Claisen–Schmidt condensation in 10% methanolic NaOH solution. Synthesis of the first intermediate was completed in 7 h at room temperature. In the next step, the second intermediate pyrazole-1-carbothioamide derivative (**2**) was obtained by processing compound (**1**) with thiosemicarbazide. Synthesis of compound (**2**) was completed in 12 h under reflux. In the last step, compound (**2**) was mixed with different phenacyl bromide derivatives in ethanol. The target compounds (**3a**–**3j**) were obtained after the reaction mixture was stirred under reflux for about 6 h. The structure of 2-[3-(3,4-dimethoxyphenyl)-5-(5-methylthiophen-2-yl)-4,5-dihydro-1*H*-pyrazol-1-yl]-4-(substitutedphenyl)­thiazoles (**3a**–**3j**) was verified using ^1^H NMR and ^13^C NMR.

**Scheme 1:**
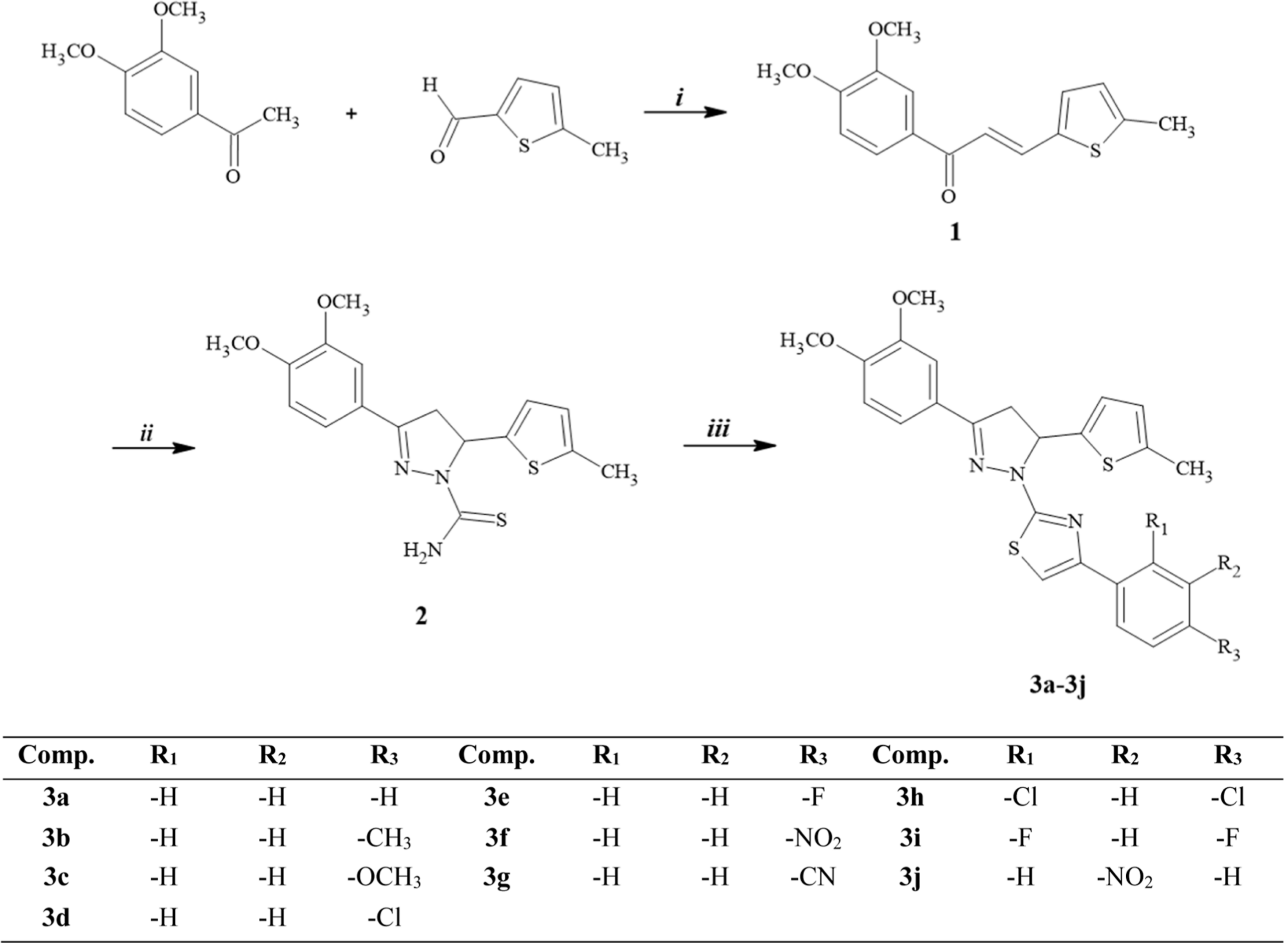
Synthetic Route for the Preparation of Compounds **3a**–**3j** ^a^

a Reagents and conditions: (i) 10% (w/v) NaOH, methanol, rt, 7 h; (ii) thiosemicarbazide, NaOH, ethanol, reflux, 12 h; (iii) substituted phenacyl bromide derivatives, ethanol, reflux, 6 h.

The signals of all the protons of compounds **3a**–**3j** were found to be in their expected region in the ^1^H NMR spectrum. The signals of the –CH_3_ proton of the 5-methylthiophen group were observed at around 2.36 ppm. The methoxy protons of 3,4-dimethoxyphenyl moiety appeared as a singlet signal in the range of 3.82–3.86 ppm. The fourth position protons of the pyrazoline ring resonated as a pair of doublets of doublets at 3.49–3.54 ppm. The fifth position proton appeared as a doublet of doublets at δ 5.88–5.93 ppm. The protons on the aromatic ring were recorded between 6.66 and 8.68 ppm. The ^13^C NMR spectra of aliphatic carbons belonging to the pyrazoline ring were observed in the range of 42.88–60.51 ppm. Methoxy (OCH_3_) carbon atoms resonated at δ: range 54.64–56.55 ppm.

### 2.2 Biological Activity

This study investigates the inhibitory potential of pyrazoline-thiazoles in modulating the catalytic activities of cholinesterase enzymes AChE and BChE, which are important targets in the therapeutic treatment of AD. The research aims to synthesize selective derivatives for cholinesterase enzymes and evaluate the efficacy of these derivatives as enzyme inhibitors and also to propose new therapeutic agents for AD through enzymatic regulation. Detailed inhibition data and findings of these derivatives are given in Table 1. The synthesized derivatives exhibited significant inhibitory activity against both AChE and BChE, operating through competitive inhibition mechanisms. Notably, the observed inhibition occurred within the low micromolar range, indicating strong binding affinities and potential efficacy as cholinesterase inhibitors. The derivatives have inhibitory activity on AD-related enzymes with IC_50_ values ranging from 0.338 to 6.417 μM, *K*_I_ values ranging from 0.045 ± 0.002 to 1.179 ± 0.083 μM for AChE, and IC_50_ values ranging from 2.087 to 7.372 μM and *K*_I_ values ranging from 0.789 ± 0.0056 to 2.374 ± 0.114 μM for BChE. Among the evaluated derivatives, compounds **3f** and **3g** demonstrated significantly higher inhibitory potential against the AChE enzyme. Specifically, these compounds against the AChE exhibited *K*_I_ values of 0.057 ± 0.003 and 0.045 ± 0.003 μM, respectively, showing inhibitory activity comparable to that of the reference compound tacrine as competitive (IC_50_: 0.046 ± 0.001 μM). All synthesized compounds, along with the positive control tacrine, exhibited competitive inhibition by binding to the active sites of both AChE and BChE. This mode of inhibition suggests that the derivatives effectively compete with the substrate for the catalytic site, highlighting their potential as active-site-directed cholinesterase inhibitors. An analysis of the enzyme kinetic parameters indicated that compounds **3f** and **3g** possess the highest selectivity for AChE. In contrast, compound **3i** displayed lower selectivity for AChE, as evidenced by its *K*_I_ values presented in Table 1. Compound **3g** also exhibited high inhibitory potential against the BChE enzyme with *K*_I_ value of 0.789 ± 0.056 μM. The inhibitory potential of this compound against both enzymes was high, and it was found to be a selective inhibitor for both enzymes.

**Table 1:** IC_50_ and *K*_I_ Values as the Inhibitory Potential of Compounds **3a**–**3j** Against AChE and BChE

Comp	AChE^a^				BChE^b^				
	IC_50_ (μM)	*R* ^2^	*K*_I_ (μM)	*R* ^2^	IC_50_ (μM)	*R* ^2^	*K*_I_ (μM)	*R* ^2^	SI^d^
**3a**	2.852	0.949	0.886 ± 0.054	0.975	4.304	0.952	1.346 ± 0.083	0.965	0.660
**3b**	4.006	0.988	0.804 ± 0.062	0.919	4.149	0.959	2.223 ± 0.132	0.974	0.361
**3c**	4.813	0.983	1.179 ± 0.083	0.943	3.066	0.947	1.160 ± 0.062	0.944	1.016
**3d**	0.523	0.909	0.121 ± 0.007	0.983	6.026	0.935	1.644 ± 0.085	0.935	0.074
**3e**	**0.385**	0.991	0.143 ± 0.084	0.961	3.316	0.962	1.724 ± 0.15	0.928	0.082
**3f**	**0.382**	0.937	0.057 ± 0.003	0.979	4.252	0.931	1.456 ± 0.071	0.945	0.039
**3g**	**0.338**	0.994	0.045 ± 0.002	0.973	**2.087**	0.952	0.789 ± 0.056	0.933	0.057
**3h**	5.457	0.919	1.105 ± 0.058	0.962	4.986	0.987	1.686 ± 0.082	0.938	0.655
**3i**	6.417	0.940	0.529 ± 0.034	0.978	7.372	0.964	1.589 ± 0.078	0.921	0.332
**3j**	4.029	0.965	0.423 ± 0.023	0.966	4.915	0.968	2.374 ± 0.114	0.918	0.178
**Tacrine** ^c^	0.150	0.983	0.046 ± 0.001	0.956	0.215	0.948	0.063 ± 0.002	0.967	0.730

a Acetylcholinesterase.

b Butyrylcholinesterase.

c Positive control.

d SI (selectivity index) = *K*_I_ (AChE)/ *K*_I_ (BChE). The best results of IC_50_, and *K*_I_ are indicated in bold.

Based on the results, a brief structure–activity relationship (SAR) can be proposed for compounds **3a**–**3j**. It seems that the substitution of *p*-cyanophenyl (compound **3g**) attached to thiazole at N1 of pyrazoline is more beneficial for the AChE inhibitory activity compared with other substituents. Besides, introducing a *p*-nitrophenyl group at the same position of the thiazole ring caused compound **3f** to be the other potent derivative after compound **3g**. The insertion of *p*-chlorophenyl (**3d**) and *p*-fluorophenyl (**3e**) substituents had a positive contribution on AChE inhibitory activity, compared to electron-donating substituents at this position (compounds **3a** and **3b**). In compounds **3c** and **3h**, the presence of *p*-methoxyphenyl and *o*,*p*-dichlorophenyl groups, respectively, led to a noticeable decrease in activity compared with other compounds. In the case of BChE inhibitory activity, it seems that introducing a *p*-cyanophenyl group attached to thiazole at N1 of the pyrazoline ring caused compound **3g** to be the most potent derivative in the series. The substitution of the *p*-methylphenyl and *m*-nitrophenyl groups (compounds **3b** and **3j**) created a dramatic decrease in the inhibition effect.

Many compound groups, including triazole derivatives, have been synthesized for cholinesterase enzymes, and their inhibitory effects on the enzyme have been screened. Medetalibeyoğlu et al. (2023) synthesized a series of novel Schiff bases containing a 1,2,4-triazole moiety and evaluated their AChE inhibitory activities. The compounds were reported to exhibit inhibitory potentials with *K*_I_ values ranging from 0.70 ± 0.07 to 8.65 ± 5.6 μM and IC_50_ values ranging from 0.43 to 3.87 μM.^47^ In a study, a series of triazole-based thiosemicarbazone derivatives were synthesized and evaluated in vitro for their AChE and BChE inhibitory activities. Most compounds were reported to exhibit inhibitory potential with IC_50_ values ranging from 0.10 ± 0.050 to 12.20 ± 0.30 μM for AChE and from 0.20 ± 0.10 to 14.10 ± 0.40 μM for BChE.^48^ In another study, various 1,2,3-triazoles were synthesized, and their inhibitory potentials against cholinesterase enzymes AChE and BChE were assessed. The inhibition studies revealed that compound 4 demonstrated AChE inhibitory potential with an IC_50_ value of 7.61 μM and a *K*_I_ value of 4.15 ± 0.75 μM, while compound 3 exhibited potential as a BChE inhibitor with an IC_50_ value of 69.30 μM and a *K*_I_ value of 60.26 ± 16.14 μM.^49^ Additionally, another research group synthesized 1,2,4-triazole derivatives and evaluated their AChE and BChE inhibitory activities. Among these, compounds 9j and 10f were found to exhibit significant inhibitory potential against AChE with IC_50_ values of 5.41 ± 0.24 and 13.57 ± 0.31 μM, respectively. Furthermore, compound 9j demonstrated strong BChE inhibition with an IC_50_ value of 7.52 ± 0.18 μM. The IC_50_ values for other compounds in the series ranged from 14.29 to 43.94 μM for AChE and from 21.59 to 41.54 μM for BChE.^50^ The derivatives synthesized in this study exhibited superior inhibitory activity against cholinesterase enzymes when compared with the compounds reported in previous studies. The compounds **3b**, **3d**, **3e**, **3f**, **3g**, **3i** and **3j** show more significant selectivity (selectivity index) for AChE than for BChE (Table 1).

### 2.3 Molecular Docking Study

Molecular modeling techniques are frequently utilized to enhance experimental data, aid in the design of bioactive and stable molecules, and determine the interaction zones of compounds with specific proteins. As a key in silico method, molecular docking serves to elucidate how small molecules bind to their target enzymes or receptors, offering insight into their biological potential. In these studies, various metrics—particularly docking scores—are used to estimate the strength and nature of these interactions, shedding light on the compounds’ bioefficacy profiles.^51–55^

The results in Table 2 show the binding affinities of ligands binding to 4EY7 and 4BDS proteins. Docking score indicates the binding free energy, and a more negative value indicates a stronger binding. This binding is due to many chemical or physical molecular interactions of the ligand with the protein, such as electrostatic, hydrophobic, and hydrogen bonds.

**Table 2:** Docking Score Values of Synthesized **3a**–**3j** derivatives and 4EY7 and 4BDS Proteins

	docking Score (kcal/mol)	
compounds	4EY7	4BDS
**3a**	–6.061	–4.774
**3b**	–6.553	–4.621
**3c**	–6.622	–4.491
**3d**	–6.726	–4.607
**3e**	–6.727	–4.668
**3f**	–6.796	–4.979
**3g**	–6.910	–5.555
**3h**	–7.166	–4.470
**3i**	–6.611	–4.716
**3j**	–6.523	–4.707
Tacrine	–9.473	–8.787

In the interaction of compound **3g** with AChE (4EY7) protein, the docking score value was found to be low (−6.910 kcal/mol), and this result corroborates with the *K*_I_ value (0.045 μM), which experimentally shows a high inhibitory effect against AChE. The in silico results of compound **3g** with high inhibitory activity for AChE experimentally show that it has a strong inhibitory effect close to the reference inhibitor tacrine. The *K*_I_ values of compounds (**3d**, **3e**, and **3f**) are also quite low (0.121, 0.143, and 0.057 μM), and the docking scores of these compounds showing interactions with the 4EY7 protein show that these compounds have a higher interaction potential with lower results than compounds **3a**, **3b**, **3c**, **3i**, and **3j**. Although the docking score and experimentally obtained *K*_I_ values often support each other, sometimes a contradiction can also be observed. For example, although compound **3h** has a low docking score value, it has a high *K*_I_ value. This is thought to indicate that factors other than the binding free energy may affect the inhibitory activity (stability of the ligand or compatibility with the protein). Compound **3g** is the ligand with the lowest *K*_I_ value, except for the reference control tacrine, making it a strong inhibitor candidate. In addition, compounds **3d**, **3e**, and **3f** ligands also stand out as strong inhibitors based on the docking score parameter findings in Table 2. These findings seem to support each other in experimental studies.

The synthesized compound **3g** has the highest activity in its interaction with the BChE (4BDS) protein, with a low docking score (−5.555 kcal/mol). This compound also has the highest inhibitory effect against BChE with an experimentally low *K*_I_ value. These findings show that modeling and experimental studies support each other. Although compound **3g** shows a lower binding affinity for 4BDS protein than tacrine, it has higher activity than the other synthesized compounds. All synthesized compounds have stronger binding affinity (more negative docking score) on 4EY7 protein compared to 4BDS. These results confirm each other with the lower *K*_I_ values obtained experimentally. The experimental and in silico results show that the synthesized compounds generally have better affinity and inhibitory activity with the 4EY7 protein. Compound **3g** stands out as the most potent inhibitor candidate for both proteins, indicating that it is more selective for the 4EY7 protein. Potent inhibitors such as compounds **3g** and **3f** can be considered as potential candidates for therapeutic applications. It is an important starting point for understanding the inhibitory effects of ligands on the 4EY7 protein. However, these results indicate that their biological efficacy needs to be studied in more depth.

As the interaction between compounds and proteins increases, bioactivity varies accordingly. 2D and 3D molecular interaction maps showing in detail the interactions that occur during the binding of ligand molecules to the active site of a protein are given in Figures 2 and 3. The 2D molecular interaction map explains the binding of ligands to the active site of the protein and shows the types of interactions that ensure binding stability.

**Figure 2:**
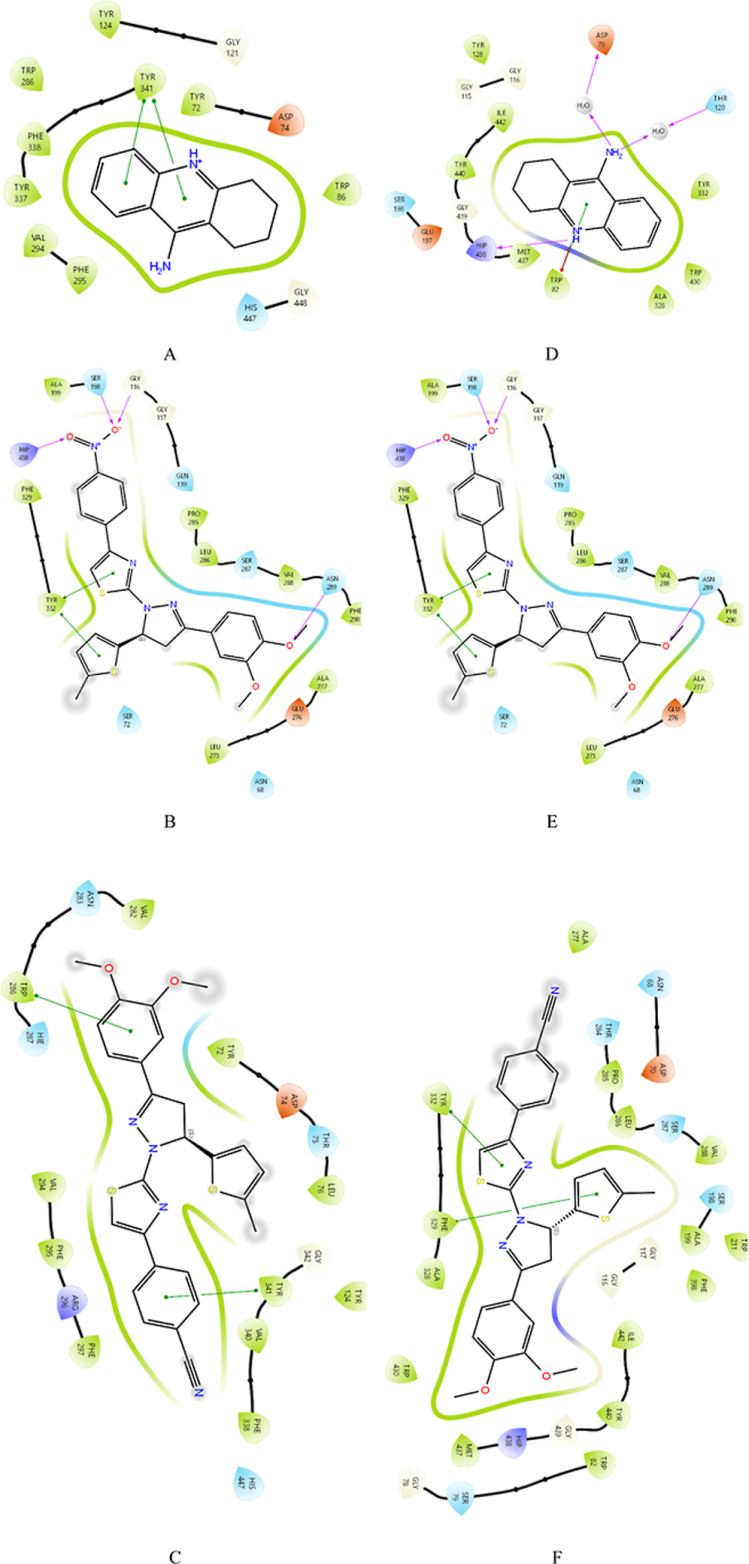
2D ligand–protein interactions between tacrine, compound **3f**, and compound **3g** with 4EY7 and 4BDS. (A) 4EY7-Tacrine, (B) 4EY7-Compound **3f**, (C) 4EY7-Compound **3g**, (D) 4BDS-Tacrine, (E) 4BDS-Compound **3f**, and (F) 4BDS-Compound **3g**.

**Figure 3:**
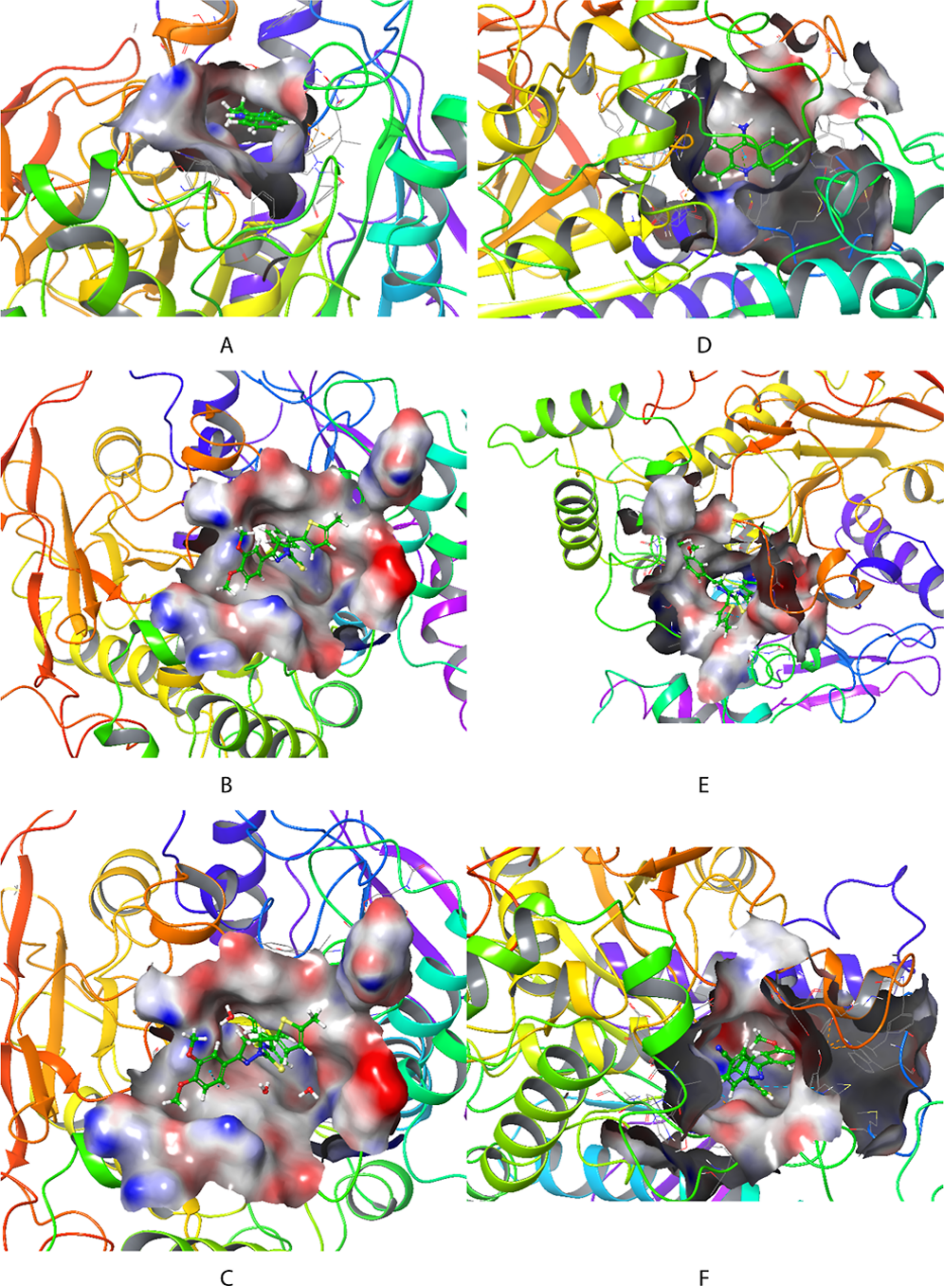
3D ligand–protein interactions between tacrine, compound **3f**, and compound **3g** with 4EY7 and 4BDS. (A) 4EY7-Tacrine, (B) 4EY7-Compound **3f**, (C) 4EY7-Compound **3g**, (D) 4BDS-Tacrine, and (E) 4BDS-Compound **3f** (F) 4BDS-Compound **3g.**

Ligands are located in a “binding pocket” in the active site of the protein. This binding pocket contains both hydrophobic and polar amino acid residues, ensuring stable binding. The type and number of interactions here are important for the binding affinity of the ligand. Aromatic rings, hydrogen bonding groups, and polar regions in the ligand structure represent important pharmacophore features that optimize binding affinity. A detailed understanding of ligand interactions in the binding site is critical for structure-based drug design (SBDD). The ligand shown here binds to the active site of the protein with strong interactions. This feature suggests that the molecule may be a potential inhibitor. In particular, hydrogen bonds, hydrophobic interactions, and van der Waals forces are some of the key factors that increase the binding affinity of the ligand. Such analyses are important in biotechnology, drug discovery, and enzyme inhibitor development.^52,62^

The amino acid residues in the 4EY7-Compound **3f** interaction show that the first oxygen atom connected to the nitrogen atom forms hydrogen bonds with SER 198 and GLY 116 and the second oxygen atom is connected to the amino acid residues HIP 438. These bonds contribute to the binding stability of the ligand. Amino acids with hydrophobic side chains such as PHE 329, TYR 332 and LEU 273 are shown to interact with the ligand. Such interactions play a critical role in ligand localization to the protein. π–π interactions between aromatic structures or cation–π interactions between the positively charged portions of the ligand and aromatic surfaces in the protein have been observed. One such interaction is demonstrated with TYR 332. Interactions such as electrostatic attraction between the negatively charged GLU 276 and the ligand play an important role in ligand binding. GLU 276 appears to be in close proximity with certain regions of the ligand. In the 4EY7-Compound **3g** interaction, π–π interactions between aromatic structures and cation–π interactions between the positively charged parts of the ligand and aromatic surfaces in the protein were observed. Such interactions were demonstrated with TRP 286 and TYR 341. Amino acids with hydrophobic properties, such as PHE 338, PHE 295, and VAL 340, show hydrophobic interactions with the ligand. These interactions favor the localization and stability of the ligand at apolar sites in the binding pocket. The negatively charged ASP74 interacts with positively charged or electrostatically attractive regions of the ligand. The interactions can increase its binding affinity.

Based on the crystal structure of AChE (PDB ID: 4EY7), the enzyme comprises two principal binding pockets: the catalytic active site (CAS) and the peripheral anionic site (PAS). The CAS includes amino acid residues such as Trp86, Tyr133, Glu202, Ser203, Tyr337, Phe338, and His447, while the PAS involves Tyr72, Asp74, Tyr124, Trp286, Phe295, and Tyr341.^56–59^ Previous research suggests that hydrophobic moieties tend to interact with Trp286 in the PAS, whereas polar or basic groups favor binding to Trp86 within the CAS.^60^ Interactions with both residues are critical for effective binding and inhibition.^61^ The studied compounds **3f** and **3g** showed strong inhibitory effects on AChE by engaging key residues in both CAS and PAS, suggesting a binding mechanism akin to that of donepezil.

In the 4BDS-Compound **3f** interaction, hydrogen bonds were formed with the first oxygen, SER 198, and GLY 116 connected to the nitrogen atom in the aromatic ring, and the second oxygen atom formed hydrogen bonds with HIP 438. On the other hand, a different hydrogen bond was formed with ASN 289. The hydrogen bond formation by HIP 438 indicates that the ligand is specifically stabilized with the protein, and this bond may play an important role. These interactions with the amino acid TYR 332, which occur between aromatic rings and hydrophobic sites, may increase the pocketability of the ligand. Interactions of regions carrying a negative charge can strengthen the binding of the ligand. In particular, GLU 276 residues can interact with certain functional groups of the ligand. In the 4BDS-Compound **3g** interaction, π–π interactions between aromatic structures or cation–π interactions between the positively charged parts of the ligand and aromatic surfaces in the protein can be observed. This type of interaction has been demonstrated with TYR 332 and PHE 329.

For a molecule to qualify as a drug candidate, it must exhibit suitable physicochemical characteristics and pharmacokinetic behavior. In silico ADME (absorption, distribution, metabolism, and excretion) predictions have gained traction in modern drug discovery due to their efficiency and cost-effectiveness.^63–65^

In the present study, various physicochemical properties, pharmacokinetic parameters, and drug-like properties of compounds **3f** and **3g** were determined through the Swiss ADME online web tool, and detailed results are shown in Table 3.

**Table 3:** Various Physicochemical Properties, Pharmacokinetic Parameters and Drug-like Properties of Compounds **3f** and **3g**

	compound **3f**	COmpound **3g**
molecular weight	506.60 g/mol	486.61 g/mol
rotatable bonds	7	6
H-bond acceptors	6	5
H-bond donors	0	0
TPSA	149.25 Å	127.22 Å
Log *P*_o/w_ (iLOGP)	3.56	4.40
GI absorption	low	low
BBB permeant	no	no
CYP1A2 inhibitor	no	no
CYP2C19 inhibitor	yes	no
CYP2C9 inhibitor	yes	yes
CYP3A4 inhibitor	yes	yes
Lipinski	yes; 0 violation	yes; 0 violation
bioavailability score	0.55	0.55
synthetic accessibility	4.55	4.56

In this study, compounds **3f** and **3g** were evaluated by using the SwissADME platform. Their molecular weights were calculated as 506.60 g/mol and 486.61 g/mol, respectively. The total polar surface areas (TPSA) were found to be 149.25 Å^2^ for 3f and 127.22 Å^2^ for 3g. Their lipophilicity, reflected by iLOGP values, was 4.40 and 3.56, respectively. The number of rotatable bonds and hydrogen bond donors/acceptors were within acceptable ranges, indicating compliance with Lipinski’s Rule of Five, a key rule-of-thumb for drug-likeness and oral bioavailability prediction. Further ADME assessments included predicted gastrointestinal absorption, blood–brain barrier permeability, and oral bioavailability estimates, reinforcing the potential of these compounds as drug candidates^64,66,67^

ADME analysis is performed in order for molecules to be used as drugs in human metabolism. Based on chemical parameters such as the molar masses of the molecules, the dipole moment of the molecules, and the hydrogen bonds given and taken by the molecules, as well as biological parameters such as the numerical values of the absorption of the molecules through the intestinal and blood barriers, absorption through the skin, or absorption through the mouth, it appears that the molecules are safe for use as drugs.^68^ Molecular docking studies have shown that potent compounds **3f** and **3g** interact with amino acid residues around the target proteins. These findings indicated that the synthesized compounds possess significant molecular and drug-like properties. Finally, the compounds may have an ideal efficacy and safety profile and therefore may be a good candidate for the development of new drugs.

In drug development processes, the physicochemical properties, pharmacokinetic parameters, and drug-likeness of candidate molecules must be thoroughly evaluated. The in silico ADME analyses of compounds **3f** and **3g** provide significant insights into their behavior in biological systems. The molecular weight of compound **3f** is 506.60 g/mol, slightly exceeding the Lipinski rule threshold, while compound **3g** has a molecular weight of 486.61 g/mol, falling within acceptable limits. This suggests that compound **3f** may face slightly more difficulty crossing the cell membranes. The TPSA values are 149.25 Å^2^ (**3f**) and 127.22 Å^2^ (**3g**), both relatively high. TPSA values above 140 Å^2^ are associated with poor oral bioavailability.^69^ Neither compound has hydrogen bond donors, which may increase the lipophilicity. The number of rotatable bonds is moderate, indicating a limited molecular flexibility. iLOGP values of 3.56 (**3f**) and 4.40 (**3g**) indicate moderate lipophilicity. However, both compounds exhibit “low” gastrointestinal (GI) absorption, likely due to their high TPSA and molecular weight. This may restrict their oral administration potential. Neither compound can permeate the BBB, which is directly associated with their high TPSA values.^70^ As such, their utility in central nervous system (CNS)-targeted therapies may be limited. Compound **3f** is predicted to inhibit the CYP2C19, CYP2C9, and CYP3A4 enzymes, whereas **3g** inhibits only CYP2C9 and CYP3A4. This suggests that compound **3g** may have a lower risk of drug–drug interactions. Inhibition of major metabolic enzymes such as CYP3A4 is clinically significant.^71^ Both compounds comply fully with Lipinski’s rules, indicating favorable drug-like profiles. Their bioavailability scores are 0.55, suggesting moderate oral bioavailability.^72^ The synthetic accessibility scores are similar (**3f**: 4.55, **3g**: 4.56), implying that both compounds are reasonably feasible to synthesize in laboratory settings.

### 2.4 Cytotoxicity Test

Cell culture studies were conducted using the previously mentioned method^73,74^ and HUVEC (healthy cell line) was grown for this study. After the cells were grown, the maximum dose accepted as 100 μM drug concentration was applied to the cell line for all compounds, and cell viability was determined.^75,76^ The results were illustrated in Table 4. According to the results, it may be suggested that even when applied at the maximum dose, none of the compounds had a decrease of more than 50% in the cell population in the healthy cell line. This shows that the synthesized compounds **3a**–**3j** were not toxic.

**Table 4:** Cell Viability (%) of HUVEC Cell Line at 100 μM Concentration for 24 h

cell Viability (%)	
**Control**	100 ± 0,50
**3a**	99,17 ± 8,75
**3b**	133,38 ± 12,88
**3c**	97,25 ± 9,97
**3d**	126,27 ± 4,11
**3e**	118,41 ± 13,05
**3f**	86,02 ± 11,34
**3g**	93,95 ± 2,88
**3h**	138,38 ± 6,96
**3i**	86,37 ± 5,54
**3j**	102,11 ± 14,53

## 3 Conclusions

By adoption of a three-step reaction strategy, diversely substituted 2-[3-(3,4-dimethoxyphenyl)-5-(5-methylthiophen-2-yl)-4,5-dihydro-1*H*-pyrazol-1-yl]-4-(substitutedphenyl)­thiazoles (**3a**–**3j**) were synthesized. NMR and HPLC techniques for purity defined each compound. The synthesized compounds were evaluated against two important drug targets for treating AD, AChE and BChE. Among them, compounds **3f** and **3g** had the most potent activity against AChE. Moreover, compound **3g** was the most potent new inhibitor against BChE. Compound **3f** had selective AChE inhibition, while compound **3g** was found to have a dual effect, being effective against both AChE and BChE. The synthesized compound **3f** has the highest activity in its interaction with the AChE (4EY7) protein with a low docking score (−6.910 kcal/mol).The synthesized compound **3g** has the highest activity in its interaction with the BChE (4BDS) protein with a low docking score (−5.555 kcal/mol). Compounds **3f** and **3g** show potential as drug candidates. However, low GI absorption and poor BBB permeability may limit their oral bioavailability and CNS-targeted use. Compound **3g** may be more favorable due to its lack of CYP2C19 inhibition. Formulation strategies aimed at enhancing the bioavailability could be considered for further development.

## 4 Materials and Methods

### 4.1 Chemistry

All of the chemicals were purchased from commercial suppliers and used without further purification. Melting points of the synthesized compounds were determined by an MP90 digital melting point apparatus (Mettler Toledo, OH, USA) and were uncorrected. ^1^H NMR and ^13^C NMR spectra of the synthesized compounds were performed by a Bruker 300 and 75 MHz digital FT-NMR spectrometer (Bruker Bioscience, Billerica, MA, USA) in DMSO-*d*_6_, respectively. All reactions were monitored by thin-layer chromatography (TLC) using Silica Gel 60 F_254_ TLC plates (Merck KGaA, Darmstadt, Germany).

#### 4.1.1 Synthesis of 1-(3,4-Dimethoxyphenyl)-3-(5-methylthiophen-2-yl)­prop-2-en-1-one (**1**)^77^

3′,4′-Dimethoxyacetophenone (3.60 g, 0.02 mol) and 5-methylthiophene-2-carbaldehyde (2.52 g, 0.02 mol) were stirred at room temperature for 7 h with 10% (w/v) aqueous sodium hydroxide solution (10 mL) in methanol (25 mL). The progress of the reaction was monitored by TLC screening. After the completion of the reaction, the precipitated compound was filtered and washed with water. The crude residue was further crystallized from ethanol.

#### 4.1.2 Synthesis of 3-(3,4-Dimethoxyphenyl)-5-(5-methylthiophen-2-yl)-4,5-dihydro-1*H*-pyrazole-1-carbothioamide (**2**)^77^

1-(3,4-Dimethoxyphenyl)-3-(5-methylthiophen-2-yl)­prop-2-en-1-one (1) (4.32 g, 0.015 mol) was reacted with thiosemicarbazide (1.37 g, 0.015 mol) and sodium hydroxide (0.72 g, 0.018 mol) in ethanol (50 mL) under reflux conditions for 12 h. The product was poured into ice-cold water. The precipitate was filtered and washed to afford compound **2**.

#### 4.1.3 Synthesis of 2-[3-(3,4-Dimethoxyphenyl)-5-(5-methylthiophen-2-yl)-4,5-dihydro-1*H*-pyrazol-1-yl]-4-(substituted phenyl)­thiazoles (**3a**–**3j**)^77^

A mixture of compound **2** (0.001 mol) and the appropriate phenacyl bromide derivatives (0.001 mol) in ethanol (30 mL) was refluxed for 6 h. After cooling, the precipitate was collected by filtration. The product was crystallized from ethanol.

##### 4.1.3.1 2-[3-(3,4-Dimethoxyphenyl)-5-(5-methylthiophen-2-yl)-4,5-dihydro-1*H*-pyrazol-1-yl]-4-phenylthiazole (**3a**)

Yield: 73%, mp = 177.2 °C. ^1^H NMR (300 MHz, DMSO-*d*_6_): δ: 2.36 (3H, s, –CH_3_), 3.50 (1H, dd, *J*_1_ = 5.40 Hz, *J*_2_ = 17.79 Hz, pyrazole C–H), 3.83 (3H, s, –OCH_3_), 3.85 (3H, s, –OCH_3_), 3.94–4.03 (1H, m, pyrazole C–H), 5.91 (1H, dd, *J*_1_ = 5.25 Hz, *J*_2_ = 11.31 Hz, pyrazole C–H), 6.66–6.67 (1H, m, Aromatic CH), 7.03–7.08 (2H, m, Aromatic CH), 7.29–7.38 (4H, m, Aromatic CH), 7.41 (2H, d, *J* = 7.23 Hz, Aromatic CH), 7.85 (2H, d, *J* = 7.11 Hz, Aromatic CH).

^13^C NMR (75 MHz, DMSO-*d*_6_): δ = 15.38 (CH_3_), 42.88 (pyrazole C), 54.64 (OCH_3_), 55.89 (OCH_3_), 60.01 (pyrazole C), 104.85, 109.42, 112.06, 120.68, 123.97, 125.23, 126.05, 126.30, 128.02, 129.03, 134.99, 136.18, 139.27, 141.97, 149.32, 150.93, 151.16, 164.14.

##### 4.1.3.2 2-[3-(3,4-Dimethoxyphenyl)-5-(5-methylthiophen-2-yl)-4,5-dihydro-1*H*-pyrazol-1-yl]-4-(4-methylphenyl)­thiazole (**3b**)

Yield: 77%, mp = 173.5 °C. ^1^H NMR (300 MHz, DMSO-*d*_6_): δ: 2.31 (3H, s, –CH_3_), 2.36 (3H, s, –CH_3_), 3.50 (1H, dd, *J*_1_ = 5.43 Hz, *J*_2_ = 17.79 Hz, pyrazole C–H), 3.83 (3H, s, –OCH_3_), 3.84 (3H, s, –OCH_3_), 3.92–4.02 (1H, m, pyrazole C–H), 5.90 (1H, dd, *J*_1_ = 5.10 Hz, *J*_2_ = 11.22 Hz, pyrazole C–H), 6.65–6.66 (1H, m, Aromatic CH), 7.02–7.03 (1H, m, Aromatic CH), 7.05–7.07 (1H, m, Aromatic CH), 7.20 (2H, d, *J* = 7.83 Hz, Aromatic CH), 7.27 (1H, s, Aromatic CH), 7.35–7.36 (2H, m, Aromatic CH), 7.73 (2H, d, *J* = 8.31 Hz, Aromatic CH).

^13^C NMR (75 MHz, DMSO-*d*_6_): δ = 15.14 (CH_3_), 21.45 (CH_3_), 43.02 (pyrazole C), 55.98 (OCH_3_), 56.55 (OCH_3_), 60.45 (pyrazole C), 103.91, 109.32, 111.99, 118.29, 120.66, 122.65, 123.89, 125.21, 125.99, 126.19, 126.31, 129.60, 132.47, 137.29, 141.92, 145.08, 151.11, 164.92.

##### 4.1.3.3 2-[3-(3,4-Dimethoxyphenyl)-5-(5-methylthiophen-2-yl)-4,5-dihydro-1*H*-pyrazol-1-yl]-4-(4-methoxyphenyl)­thiazole (**3c**)

Yield: 67%, mp = 197.2 °C. ^1^H NMR (300 MHz, DMSO-*d*_6_): δ: 2.36 (3H, s, –CH_3_), 3.52 (1H, dd, *J*_1_ = 4.59 Hz, *J*_2_ = 17.94 Hz, pyrazole C–H), 3.78 (3H, s, –OCH_3_), 3.82 (3H, s, –OCH_3_), 3.84 (3H, s, –OCH_3_), 3.92–4.02 (1H, m, pyrazole C–H), 5.89 (1H, dd, *J*_1_ = 5.28 Hz, *J*_2_ = 11.34 Hz, pyrazole C–H), 6.65–6.66 (1H, m, Aromatic CH), 6.96 (2H, d, *J* = 8.91 Hz, Aromatic CH), 7.02–7.07 (2H, m, Aromatic CH), 7.18 (1H, s, Aromatic CH), 7.31–7.36 (2H, m, Aromatic CH), 7.77 (2H, d, *J* = 8.82 Hz, Aromatic CH).

^13^C NMR (75 MHz, DMSO-*d*_6_): δ = 15.45 (CH_3_), 43.26 (pyrazole C), 55.60 (OCH_3_), 55.98 (OCH_3_), 56.07 (OCH_3_), 60.39 (pyrazole C), 102.65, 103.04, 106.42, 106.85, 109.31, 112.00, 114.37, 120.64, 122.90, 123.97, 125.21, 126.27, 127.37, 127.88, 135.59, 141.79, 145.75, 153.42.

##### 4.1.3.4 2-[3-(3,4-Dimethoxyphenyl)-5-(5-methylthiophen-2-yl)-4,5-dihydro-1*H*-pyrazol-1-yl]-4-(4-chlorophenyl)­thiazole (**3d**)

Yield: 62%, mp = 175.2 °C. ^1^H NMR (300 MHz, DMSO-*d*_6_): δ: 2.36 (3H, s, –CH_3_), 3.52 (1H, dd, *J*_1_ = 4.59 Hz, *J*_2_ = 17.94 Hz, pyrazole C–H), 3.83 (3H, s, –OCH_3_), 3.85 (3H, s, –OCH_3_), 3.93–4.03 (1H, m, pyrazole C–H), 5.91 (1H, dd, *J*_1_ = 4.38 Hz, *J*_2_ = 10.59 Hz, pyrazole C–H), 6.66–6.67 (1H, m, Aromatic CH), 7.03–7.08 (2H, m, Aromatic CH), 7.36 (1H, s, Aromatic CH), 7.43–7.49 (3H, m, Aromatic CH), 7.87 (3H, dd, *J*_1_ = 1.68 Hz, *J*_2_ = 8.61 Hz, Aromatic CH).

^13^C NMR (75 MHz, DMSO-*d*_6_): δ = 15.43 (CH_3_), 43.13 (pyrazole C), 55.99 (OCH_3_), 56.16 (OCH_3_), 60.15 (pyrazole C), 105.20, 109.30, 112.01, 114.76, 117.55, 120.73, 121.21, 124.16, 125.25, 126.32, 127.73, 128.17, 129.09, 132.69, 136.35, 139.23, 142.56, 153.76.

##### 4.1.3.5 2-[3-(3,4-Dimethoxyphenyl)-5-(5-methylthiophen-2-yl)-4,5-dihydro-1*H*-pyrazol-1-yl]-4-(4-fluorophenyl)­thiazole (**3e**)

Yield: 70%, mp = 153.9 °C. ^1^H NMR (300 MHz, DMSO-*d*_6_): δ: 2.35 (3H, s, –CH_3_), 3.50 (1H, dd, *J*_1_ = 5.40 Hz, *J*_2_ = 17.82 Hz, pyrazole C–H), 3.82 (3H, s, –OCH_3_), 3.84 (3H, s, –OCH_3_), 3.92–4.02 (1H, m, pyrazole C–H), 5.90 (1H, dd, *J*_1_ = 5.19 Hz, *J*_2_ = 11.31 Hz, pyrazole C–H), 6.64–6.66 (1H, m, Aromatic CH), 7.02–7.07 (2H, m, Aromatic CH), 7.21–7.24 (2H, m, Aromatic CH), 7.34–7.35 (2H, m, Aromatic CH), 7.87–7.90 (3H, m, Aromatic CH).

^13^C NMR (75 MHz, DMSO-*d*_6_): δ = 15.44 (CH_3_), 43.23 (pyrazole C), 55.97 (OCH_3_), 56.06 (OCH_3_), 60.21 (pyrazole C), 104.56, 109.31, 111.53, 111.98, 115.75, 116.04, 120.69, 123.89, 125.23, 126.30, 127.94, 128.05, 131.42, 139.17, 141.91, 149.83, 153.75, 160.45.

##### 4.1.3.6 2-[3-(3,4-Dimethoxyphenyl)-5-(5-methylthiophen-2-yl)-4,5-dihydro-1*H*-pyrazol-1-yl]-4-(4-nitrophenyl)­thiazole (**3f**)

Yield: 78%, mp = 169.3 °C. ^1^H NMR (300 MHz, DMSO-*d*_6_): δ: 2.36 (3H, s, –CH_3_), 3.54 (1H, dd, *J*_1_ = 5.25 Hz, *J*_2_ = 17.91 Hz, pyrazole C–H), 3.83 (3H, s, –OCH_3_), 3.85 (3H, s, –OCH_3_), 3.95–4.04 (1H, m, pyrazole C–H), 5.93 (1H, dd, *J*_1_ = 5.10 Hz, *J*_2_ = 11.22 Hz, pyrazole C–H), 6.66–6.67 (1H, m, Aromatic CH), 7.08–7.09 (2H, m, Aromatic CH), 7.36 (1H, s, Aromatic CH), 7.75 (1H, s, Aromatic CH), 8.08–8.12 (2H, m, Aromatic C–H), 8.26–8.31 (3H, m, Aromatic C–H).

^13^C NMR (75 MHz, DMSO-*d*_6_): δ = 15.43 (CH_3_), 43.51 (pyrazole C), 55.99 (OCH_3_), 56.08 (OCH_3_), 60.51 (pyrazole C), 109.38, 109.74, 112.01, 116.78, 117.69, 120.80, 123.77, 124.59, 125.29, 126.50, 126.83, 139.43, 140.99, 146.69, 149.29, 151.24, 154.14, 179.59.

##### 4.1.3.7 2-[3-(3,4-Dimethoxyphenyl)-5-(5-methylthiophen-2-yl)-4,5-dihydro-1*H*-pyrazol-1-yl]-4-(4-cyanophenyl)­thiazole (**3g**)

Yield: 67%, mp = 164.4 °C. ^1^H NMR (300 MHz, DMSO-*d*_6_): δ: 2.36 (3H, s, –CH_3_), 3.49 (1H, dd, *J*_1_ = 5.34 Hz, *J*_2_ = 18.12 Hz, pyrazole C–H), 3.84 (3H, s, –OCH_3_), 3.86 (3H, s, –OCH_3_), 3.95–4.05 (1H, m, pyrazole C–H), 5.91–5.94 (1H, m, pyrazole C–H), 6.66–6.67 (1H, m, Aromatic CH), 7.04–7.09 (2H, m, Aromatic CH), 7.33–7.37 (2H, m, Aromatic C–H), 7.67–7.68 (1H, m, Aromatic CH), 7.88 (2H, dd, *J*_1_ = 2.88 Hz, *J*_2_ = 8.49 Hz, Aromatic C–H), 8.02 (2H, dd, *J*_1_ = 2.7 Hz, *J*_2_ = 8.34 Hz, Aromatic C–H).

^13^C NMR (75 MHz, DMSO-*d*_6_): δ = 15.44 (CH_3_), 43.28 (pyrazole C), 55.98 (OCH_3_), 56.08 (OCH_3_), 60.17 (pyrazole C), 108.71, 109.39, 110.06, 112.02, 119.51, 120.61, 120.78, 123.80, 125.27, 126.38, 126.60, 133.18, 139.09, 139.38, 141.59, 149.20, 151.23, 154.03, 164.85.

##### 4.1.3.8 2-[3-(3,4-Dimethoxyphenyl)-5-(5-methylthiophen-2-yl)-4,5-dihydro-1*H*-pyrazol-1-yl]-4-(2,4-dichlorophenyl)­thiazole (**3h**)

Yield: 61%, mp = 143.3 °C. ^1^H NMR (300 MHz, DMSO-*d*_6_): δ: 2.36 (3H, s, –CH_3_), 3.49 (1H, dd, *J*_1_ = 5.31 Hz, *J*_2_ = 17.85 Hz, pyrazole C–H), 3.82 (3H, s, –OCH_3_), 3.84 (3H, s, –OCH_3_), 3.93–4.02 (1H, m, pyrazole C–H), 5.88 (1H, dd, *J*_1_ = 5.19 Hz, *J*_2_ = 11.22 Hz, pyrazole C–H), 6.64–6.65 (1H, m, Aromatic CH), 6.98–6.99 (2H, m, Aromatic CH), 7.06 (1H, dd, *J*_1_ = 1.56 Hz, *J*_2_ = 8.28 Hz, Aromatic C–H), 7.32–7.36 (2H, m, Aromatic C–H), 7.49–7.53 (2H, m, Aromatic CH), 7.67–7.68 (1H, m, Aromatic CH), 7.91 (1H, dd, *J*_1_ = 1.77 Hz, *J*_2_ = 8.55 Hz, Aromatic C–H).

^13^C NMR (75 MHz, DMSO-*d*_6_): δ = 15.44 (CH_3_), 43.39 (pyrazole C), 55.99 (OCH_3_), 56.08 (OCH_3_), 60.23 (pyrazole C), 105.27, 109.36, 110.56, 112.00, 120.74, 123.84, 125.24, 126.29, 127.99, 130.28, 131.83, 132.27, 132.68, 132.88, 139.27, 141.86, 146.24, 149.28, 151.19, 153.87.

##### 4.1.3.9 2-[3-(3,4-Dimethoxyphenyl)-5-(5-methylthiophen-2-yl)-4,5-dihydro-1*H*-pyrazol-1-yl]-4-(2,4-difluorophenyl)­thiazole (**3i**)

Yield: 78%, mp = 152.8 °C. ^1^H NMR (300 MHz, DMSO-*d*_6_): δ: 2.35 (3H, s, –CH_3_), 3.50 (1H, dd, *J*_1_ = 5.43 Hz, *J*_2_ = 17.85 Hz, pyrazole C–H), 3.82 (3H, s, –OCH_3_), 3.84 (3H, s, –OCH_3_), 3.93–4.02 (1H, m, pyrazole C–H), 5.89 (1H, dd, *J*_1_ = 5.22 Hz, *J*_2_ = 11.25 Hz, pyrazole C–H), 6.64–6.66 (1H, m, Aromatic CH), 7.02–7.07 (2H, m, Aromatic CH), 7.18 (1H, dd, *J*_1_ = 2.46 Hz, *J*_2_ = 8.22 Hz, Aromatic C–H), 7.21–7.22 (1H, m, Aromatic C–H), 7.30–7.37 (3H, m, Aromatic CH), 8.03 (1H, dd, *J*_1_ = 1.92 Hz, *J*_2_ = 8.85 Hz, Aromatic C–H).

^13^C NMR (75 MHz, DMSO-*d*_6_): δ = 15.42 (CH_3_), 43.28 (pyrazole C), 55.98 (OCH_3_), 56.07 (OCH_3_), 60.18 (pyrazole C), 109.35, 110.34, 111.99, 112.17, 113.06, 119.63, 120.73, 123.79, 123.86, 125.24, 126.32, 127.46, 129.93, 139.33, 141.86, 143.72, 149.29, 151.19, 153.85, 164.34.

##### 4.1.3.10 2-[3-(3,4-Dimethoxyphenyl)-5-(5-methylthiophen-2-yl)-4,5-dihydro-1*H*-pyrazol-1-yl]-4-(3-nitrophenyl)­thiazole (**3j**)

Yield: 62%, mp = 172.9 °C. ^1^H NMR (300 MHz, DMSO-*d*_6_): δ: 2.37 (3H, s, –CH_3_), 3.54 (1H, dd, *J*_1_ = 5.82 Hz, *J*_2_ = 17.88 Hz, pyrazole C–H), 3.83 (3H, s, –OCH_3_), 3.85 (3H, s, –OCH_3_), 3.95–4.04 (1H, m, pyrazole C–H), 5.91 (1H, dd, *J*_1_ = 5.67 Hz, *J*_2_ = 11.40 Hz, pyrazole C–H), 6.67–6.68 (1H, m, Aromatic CH), 7.05–7.08 (2H, m, Aromatic CH), 7.33–7.36 (2H, m, Aromatic CH), 7.68–7.73 (2H, m, Aromatic CH), 8.14 (1H, dd, *J*_1_ = 2.28 Hz, *J*_2_ = 8.13 Hz, Aromatic C–H), 8.27 (1H, d, *J* = 7.77 Hz, Aromatic C–H), 8.67–8.68 (1H, m, Aromatic CH).

^13^C NMR (75 MHz, DMSO-*d*_6_): δ = 15.42 (CH_3_), 43.26 (pyrazole C), 55.98 (OCH_3_), 56.07 (OCH_3_), 60.27 (pyrazole C), 107.59, 109.37, 111.99, 120.58, 120.76, 122.55, 123.80, 125.25, 126.38, 130.67, 132.06, 136.55, 139.44, 141.69, 148.54, 148.73, 149.28, 151.21, 154.02, 165.19.

### 4.2 Cholinesterase Activity Assay

To evaluate cholinesterase activity, a modified version of the method described by Ellman et al. (1961) was employed.^78^ Acetylthiocholine iodide (ATChI) and butyrylcholine iodide (BChI) were used as substrates for AChE (from electric eel) and BChE (from equine serum), respectively, with activity changes monitored at 37 °C. The assay was conducted in a cuvette containing Tris–HCl buffer (50 mM; pH 8.0), DTNB solution (0.01 M, 100 μL), and ATChI/BChI solutions (0.050 M, 50 μL), followed by the addition of the enzyme solution (5.32 × 10^–3^ U, 50 μL). After a 5 min incubation period, absorbance measurements were taken at 412 nm using a spectrophotometer, with each assay performed in triplicate.^79^

### 4.3 In vitro Inhibition Studies

Stock solutions of the inhibitor compounds were prepared in 20% DMSO. The inhibition observed with 20% DMSO alone (used as the negative control) was subtracted from the total inhibition values, ensuring that only the inhibition due to the tested compounds was considered in the analysis. To assess cholinesterase activity inhibition ranging from 0% to 100%, triazole derivatives were evaluated at a minimum of five different inhibitor concentrations, typically within the range of 10^–2^ to 10^2^ μM. The inhibitory effects of compounds **3a**–**3j** were evaluated across various concentrations of inhibitors, utilizing at least five distinct concentrations against AChE and BChE enzymes. The IC_50_ values for the derivatives were derived from the plots of activity percentage versus inhibitor concentration. Additionally, inhibition types and inhibition constants (*K*_I_) were determined through Lineweaver–Burk plots, thus providing comprehensive data on the kinetic behavior of these inhibitors.^80,81^

### 4.4 Molecular Docking Study

Acetylcholinesterase (AChE) and butyrylcholinesterase (BChE) are critical enzymatic targets in the treatment of neurodegenerative disorders, particularly Alzheimer’s disease. Given the abundance of crystallographic structures of these enzymes available in the Protein Data Bank (PDB), the selection of suitable structures for molecular docking studies is of paramount importance. In the present study, PDB ID: 4EY7 was selected for AChE and PDB ID: 4BDS for BChE based on several key criteria. Both structures offer high-resolution data 2.35 Å for 4EY7 and 2.10 Å for 4BDS, which ensures precise and detailed modeling of the active sites.^82,83^ Importantly, these crystal structures represent the holo forms of the enzymes, each cocrystallized with a known inhibitor: donepezil in 4EY7 and a hybrid inhibitor in 4BDS. This provides a realistic conformational state of the binding pocket, which is crucial for the accuracy of docking simulations.^84,85^

The docking studies were conducted using structural data from the Protein Data Bank, focusing on AChE and butyrylcholinesterase (BChE). Schrödinger’s Maestro software suite was used throughout, utilizing modules such as the Protein Preparation Wizard for receptor optimization and LigPrep for ligand refinement.^51,86,87^ Glide was employed for docking simulations to analyze binding poses and affinity scores. Additionally, QikProp was used to predict ADME/T characteristics, offering a comprehensive view of the compounds’ expected in vivo behaviors.^52,62,88,89^

### 4.5 ADME Analysis

Canonical SMILES representations for each compound were created using ChemDraw and subsequently analyzed using SwissADME (http://www.swissadme.ch/). Parameters such as lipophilicity, polar surface area, rotatable bonds, and Lipinski rule compliance were evaluated. These outputs provided meaningful predictions on bioavailability and pharmacokinetic suitability, bolstering the value of compounds **3f** and **3g** as leads in the drug development pipeline.

### 4.6 Statistical Studies

The data analysis and graphical representations were performed using GraphPad Prism version 8 for Windows (GraphPad Software, La Jolla, California, USA), a widely utilized software known for its comprehensive statistical tools and intuitive interface. In biological experiments (enzyme inhibition, etc.), measurements were performed in 3 independent replicates. Descriptive statistical analyses were carried out, with the results presented as means ± standard error of the mean (SEM), offering an indication of the variability associated with the mean values.

### 4.7 Cytotoxicity Test

The cytotoxic effect of all compounds by MTT analysis was performed to determine the proliferation on the HUVEC cell line. The MTT [3-(4,5-dimethyl-2-thiazol)-2,5-diphenyl-2*H*-tetrazolium bromide] cytotoxicity assay was employed according to previous studies with minor modifications.^70–73^

## Supplementary Material



## Notes

^1^H NMR, ^13^C NMR, and HRMS spectra for all compounds (PDF)

## References

[1] Kumar S., Mahajan A., Ambatwar R., Khatik G. L. (2024). Recent advancements
in the treatment of alzheimer’s disease: a multitarget-directed
ligand approach. Curr. Med. Chem..

[2] Türkeş C., Akocak S., Işık M., Lolak N., Taslimi P., Durgun M., Gülçin I. ˙., Budak Y., Beydemir S. ¸. (2022). Novel
inhibitors with sulfamethazine
backbone: synthesis and biological study of multi-target cholinesterases
and α-glucosidase inhibitors. J. Biomol.
Struct. Dyn..

[3] Xiao D., Zhang C. (2024). Current therapeutics
for Alzheimer’ s disease and clinical
trials. Explor. Neurosci..

[4] O’Brien R. J., Wong P. C. (2011). Amyloid precursor protein processing and Alzheimer’s
disease. Annu. Rev. Neurosci..

[5] Medeiros R., Baglietto-Vargas D., LaFerla F. M. (2011). The role of tau in Alzheimer’s
disease and related disorders. CNS Neurosci.
Ther.

[6] Baumgartner T. J., Haghighijoo Z., Goode N. A., Dvorak N. M., Arman P., Laezza F. (2023). Voltage-gated
Na­(+) channels in Alzheimer’s
disease: physiological roles and therapeutic potential. Life.

[7] Mueed Z., Tandon P., Maurya S. K., Deval R., Kamal M. A., Poddar N. K. (2019). Tau and mTOR: The Hotspots for Multifarious
Diseases
in Alzheimer’s Development. Front. Neurosci..

[8] Işık M., Beydemir S. (2022). AChE mRNA expression as a possible
novel biomarker
for the diagnosis of coronary artery disease and Alzheimer’s
disease, and its association with oxidative stress. Arch. Physiol. Biochem..

[9] Chen Z. R., Huang J. B., Yang S. L., Hong F. F. (2022). Role of cholinergic
signaling in Alzheimer’s disease. Molecules.

[10] Patel D. V., Patel N. R., Kanhed A. M., Teli D. M., Patel K. B., Gandhi P. M., Patel S. P., Chaudhary B. N., Shah D. B., Prajapati N. K., Patel K. V., Yadav M. R. (2020). Further
studies on triazinoindoles as potential novel multitarget-directed
anti-Alzheimer’s agents. ACS Chem. Neurosci..

[11] Elsawalhy M., Abdel-Rahman A. A., Basiony E. A., Ellithy S. A., Hassan A. A., Abou-Amra E. S., Ismail A., Almehizia A. A., Al-Omar M. A., Naglah A. M. (2024). Novel dual acetyl-and
butyrylcholinesterase inhibitors based on the pyridyl–pyridazine
moiety for the potential treatment of Alzheimer’s disease. Pharmaceuticals.

[12] Giacobini E. (2003). Cholinesterases:
new roles in brain function and in Alzheimer’s disease. Neurochem. Res..

[13] Mesulam M. M., Geula C. (1994). Butyrylcholinesterase
reactivity differentiates the amyloid plaques
of aging from those of dementia. Ann. Neurol..

[14] Makhaeva G. F., Kovaleva N. V., Boltneva N. P., Lushchekina S. V., Astakhova T. Y., Rudakova E. V., Proshin A. N., Serkov I. V., Radchenko E. V., Palyulin V. A., Bachurin S. O., Richardson R. J. (2020). New hybrids
of 4-amino-2, 3-polymethylene-quinoline and p-tolylsulfonamide as
dual inhibitors of acetyl-and butyrylcholinesterase and potential
multifunctional agents for Alzheimer’s disease treatment. Molecules.

[15] Miao S., He Q., Li C., Wu Y., Liu M., Chen Y., Qi S., Gong K. (2022). Aaptamine
- a dual acetyl - and butyrylcholinesterase
inhibitor as potential anti-Alzheimer’s disease agent. Pharm. Biol..

[16] Bondžić A. M., Senćanski M. V., Vujačić Nikezić A. V., Kirillova M. V., André V., Kirillov A. M., Bondžić B. P. (2020). Aminoalcoholate-driven
tetracopper­(II) cores as dual acetyl and butyrylcholinesterase inhibitors:
Experimental and theoretical elucidation of mechanism of action. J. Inorg. Biochem..

[17] Zhang J., Zhang Y., Wang J., Xia Y., Zhang J., Chen L. (2024). Recent advances in Alzheimer’s
disease: Mechanisms, clinical
trials and new drug development strategies. Signal Transduct. Target. Ther..

[18] Moss D. E., Perez R. G., Kobayashi H. (2016). Cholinesterase
inhibitor therapy
in Alzheimer’s disease: the limits and tolerability of irreversible
CNS-selective acetylcholinesterase inhibition in primates. J. Alzheimers Dis..

[19] Singh K., Pal R., Khan S. A., Kumar B., Akhtar M. J. (2021). Insights into the
structure activity relationship of nitrogen-containing heterocyclics
for the development of antidepressant compounds: An updated review. J. Mol. Struct..

[20] Gok S., Murat Demet M., Özdemir A., Turan-Zitouni G. (2010). Evaluation
of antidepressant-like effect of 2-pyrazoline derivatives. Med. Chem. Res..

[21] Nasab N. H., Azimian F., Eom Y. S., Shah F. H., Kim S. J. (2023). Synthesis,
anticancer evaluation, and molecular docking studies of thiazolyl-pyrazoline
derivatives. Bioorg. Med. Chem. Lett..

[22] Salih R. H. H., Hasan A. H., Hussen N. H., Hawaiz F. E., Hadda T. B., Jamalis J., Almalki F. A., Adeyinka A. S., Coetzee L. C. C., Oyebamiji A. K. (2023). Thiazole-pyrazoline hybrids as potential
antimicrobial
agent: Synthesis, biological evaluation, molecular docking, DFT studies
and POM analysis. J. Mol. Struct..

[23] Singh H., Kumar R., Mazumder A., Salahuddin, Kumar
Yadav R., Kukreti N., Abdullah M. M., Kumar
Tyagi P., Chaitanya M. V. N. L. (2024). Synthesis, in vivo, and in silico
evaluation of new
pyrazoline-benzothiazole conjugates as antiepileptic agents. Chem. Biodivers..

[24] Najmuldeen Z. D., Omar T. N. (2023). Synthesis, characterization and evaluation of new pyrazoline
derivatives containing sulfonamide moiety as anti-microbial and anti-inflammatory
agents. J. Res. Med. Dent..

[25] de
Farias I. F., de Oliveira Gonçalves R., de Freitas M. D., da Silva F. L., de Sousa A. F., Trevisan M. T. S., Lomonaco D., Monte F. J. Q., da Fonseca A. M., de Lemos T. L. G. (2023). Synthesis, characterization and molecular docking study
of pyrazolines synthesized from chalcones: Antioxidant and acetylcholinesterase
activities. J. Mol. Struct..

[26] Tuğrak M., Gül H. I. ˙., Gülçin I. ˙. (2020). Acetylcholinesterase
inhibitory potencies of new pyrazoline derivatives. J. Res. Pharm..

[27] Selatnia I., Khamaysa O. M. A., Soliman A. G., Bourzami R., Sid A., Lgaz H., Mokhnache K., Alrashdi A. A., Bensouici C. (2024). Mechanistic
insights and therapeutic implications of novel pyrazoline derivatives
on antioxidant and enzymatic inhibitory activities. J. Mol. Struct..

[28] Hussein A. J. (2024). Synthesis,
antimicrobial and antioxidant activity of some new pyrazolines containing
azo linkages. Curr. Org. Synth..

[29] Abou-Zied H. A., Beshr E. A. M., Hayallah A. M., Abdel-Aziz M. (2024). Emerging insights
into pyrazoline motifs: A comprehensive exploration of biological
mechanisms and prospects for future advancements. J. Mol. Struct..

[30] Ahsan M. J., Ali A., Ali A., Thiriveedhi A., Bakht M. A., Yusuf M., Salahuddin A. O., Afzal O., Altamimi A. S. A. (2022). Pyrazoline containing
compounds as therapeutic targets for neurodegenerative disorders. ACS Omega.

[31] Chauhan A., Salahuddin S., Mazumder A., Kumar R., Jawed Ahsan M., Shahar Yar M., Maqsood R., Singh K. (2024). Targeted development
of pyrazoline derivatives for neurological disorders: a review. ChemistrySelect.

[32] Altıntop M. D., Sağlık Özkan B. N., Özdemir A. (2024). Design, synthesis,
and evaluation of new pyrazolines as small molecule inhibitors of
acetylcholinesterase. ACS omega.

[33] Uğraş Z., Tok F., Çakir C., Tuna K., Tatar-Yilmaz G., Mutlu D., Sıcak Y., Arslan S. ¸., Öztürk M., Koçyiğit-Kaymakçioğlu B. (2024). Exploring
2-pyrazoline derivatives as potent antidiabetic agents and cholinesterase
inhibitors: their synthesis and molecular docking studies. J. Mol. Struct..

[34] Machado V., Cenci A. R., Teixeira K. F., Sens L., Tizziani T., Nunes R. J., Ferreira L. L. G., Yunes R. A., Sandjo L. P., Andricopulo A. D., de Oliveira A. S. (2022). Pyrazolines as potential anti-Alzheimer’s
agents: DFT, molecular docking, enzyme inhibition and pharmacokinetic
studies. RSC Med. Chem..

[35] Sakarya M. T., Gul H. I., Yamali C., Ozkay Y., Tok T. T. (2024). In vitro
enzyme activity and molecular docking studies of pyrazoline derivatives
as monoamine oxidase inhibitors. Pharm. Chem.
J..

[36] Ozgun D. O., Gul H. I., Yamali C., Sakagami H., Gulcin I., Sukuroglu M., Supuran C. T. (2019). Synthesis and bioactivities of pyrazoline
benzensulfonamides as carbonic anhydrase and acetylcholinesterase
inhibitors with low cytotoxicity. Bioorg. Chem..

[37] Sever B., Türkeş C., Altıntop M. D., Demir Y., Beydemir S. ¸. (2020). Thiazolyl-pyrazoline
derivatives: In vitro and in silico evaluation as potential acetylcholinesterase
and carbonic anhydrase inhibitors. Int. J. Biol.
Macromol..

[38] Machado V., Cenci A. R., Teixeira K. F., Sens L., Tizziani T., Nunes R. J., Ferreira L. L. G., Yunes R. A., Sandjo L. P., Andricopulo A. D., de Oliveira A. S. (2022). Pyrazolines as potential anti-Alzheimer’s
agents: DFT, molecular docking, enzyme inhibition and pharmacokinetic
studies. RSC Med. Chem..

[39] Obaid R. J., Naeem N., Mughal E. U., Al-Rooqi M. M., Sadiq A., Jassas R. S., Moussa Z., Ahmed S. A. (2022). Inhibitory potential
of nitrogen, oxygen and sulfur containing heterocyclic scaffolds against
acetylcholinesterase and butyrylcholinesterase. RSC Adv..

[40] Sever B., Türkeş C., Altıntop M. D., Demir Y., Akalın
Çiftçi G., Beydemir S. (2021). Novel metabolic enzyme inhibitors
designed through the molecular hybridization of thiazole and pyrazoline
scaffolds. Arch. Pharm..

[41] Temel H. E., Altintop M. D., Özdemir A. (2018). Synthesis and evaluation of a new
series of thiazolyl-pyrazoline derivatives as cholinesterase inhibitors. Turk. J. Pharm. Sci..

[42] Unsal-Tan O., Tüylü Küçükkılınç T., Ayazgök B., Balkan A., Ozadali-Sari K. (2019). Synthesis,
molecular docking, and biological evaluation of novel 2-pyrazoline
derivatives as multifunctional agents for the treatment of Alzheimer’s
disease. MedChemComm.

[43] Bajad N. G., Jangra J., Kumar A., Krishnamurthy S., Singh S. K. (2025). Discovery of pyrazoline analogs as multi-targeting
cholinesterase, β-secretase and Aβ aggregation inhibitors
through lead optimization strategy. Int. J.
Biol. Macromol..

[44] Zhang Z. Y., Han S. T., Mingyu W., Zien Y., Hu P. H., He R., Cao Y. Y., Shi D. H. (2025). Synthesis and characterization of
thiazole derivatives as cholinesterase inhibitors. ChemistrySelect.

[45] Jehangir U., Khan S., Hussain R., Khan Y., Rahim F., Iqbal T., Aziz T., Alasmari A. F. (2024). In vitro and in
silico correlation of bis-thiazole based Schiff base hybrids analogues:
A computational approach develop to promising acetylcholinesterase
and butyrylcholinesterase inhibitors. J. Mol.
Struct..

[46] Hussain F., Tahir A., Rehman H. M., Wu Y., Shah M., Rashid U. (2025). Promising thiazolidinedione-thiazole
based multi-target
and neuroprotective hybrids for Alzheimer’s disease: Design,
synthesis, in-vitro, in-vivo and in-silico studies. Eur. J. Med. Chem..

[47] Medetalibeyoğlu H., Türkan F., Manap S., Bursal E., Beytur M., Aras A., Akyıldırım O., Kotan G., Gürsoy-Kol O.
¨., Yüksek H. (2023). Synthesis
and acetylcholinesterase enzyme inhibitory effects of some novel 4,5-Dihydro-1H-1,2,4-triazol-5-one
derivatives; an in vitro and in silico study. J. Biomol. Struct. Dyn..

[48] Rahim F., Ullah H., Taha M., Hussain R., Sarfraz M., Iqbal R., Iqbal N., Khan S., Ali Shah S. A., Albalawi M. A., Abdelaziz M. A., Alatawi F. S., Alasmari A., Sakran M. I., Zidan N., Jafri I., Khan K. M. (2023). Synthesis
of new triazole-based thiosemicarbazone derivatives as anti-Alzheimer’s
disease candidates: evidence-based in vitro study. Molecules.

[49] Almaz Z. (2023). Investigation
of biological activities of various 1,2,3-triazole compounds: Their
effects on cholinesterase enzymes, determination of antioxidant capacity
and antimicrobial activity. J. Biochem. Mol.
Toxicol..

[50] Riaz N., Iftikhar M., Saleem M., Aziz ur R., Hussain S., Rehmat F., Afzal Z., Khawar S., Ashraf M., al-Rashida M. (2020). New synthetic
1,2,4-triazole derivatives: Cholinesterase
inhibition and molecular docking studies. Results
Chem..

[51] Singh A., Sharma S., Arora S., Attri S., Kaur P., Kaur Gulati H., Bhagat K., Kumar N., Singh H., Vir Singh J., Mohinder Singh Bedi P. (2020). New coumarin-benzotriazole based
hybrid molecules as inhibitors of acetylcholinesterase and amyloid
aggregation. Bioorg. Med. Chem. Lett..

[52] Schrödinger Release 2021–3: protein preparation wizard. Epik, Schrödinger, LLC/Impact, Schrödinger, LLC/Prime, Schrödinger, LLC, (NY)/New York (NY)/New York (NY): New York; 2021.

[53] Durgun M., Akocak S., Lolak N., Topal F., Koçyiğit Ü. M., Türkeş C., Işık M., Beydemir Ş. (2024). Design and synthesis
of pyrazole carboxamide derivatives
as selective cholinesterase and carbonic anhydrase inhibitors: molecular
docking and biological evaluation. Chem. Biodivers..

[54] Amangeldinova M., Ersatır M., Necip A., Yilmaz M. A., Cimentepe M., Kudrina N., Terletskaya N. V., Ozturk Cimentepe O., Yildirim M. (2024). Simultaneous quantitative screening
of 53 phytochemicals
from Rheum tataricum L. roots: a comparative study of supercritical
CO2, subcritical ethanol, and ultrasound-assisted extraction for enhanced
antioxidant, antibacterial activities, and molecular docking study. Front. Plant Sci..

[55] Lolak N., Akocak S., Durgun M., Duran H. E., Necip A., Türkeş C., Işık M., Beydemir S. ¸. (2023). Novel bis-ureido-substituted
sulfaguanidines and sulfisoxazoles as carbonic anhydrase and acetylcholinesterase
inhibitors. Mol. Divers..

[56] Colovic M. B., Krstic D., Lazarevic-Pactil T., Bondzic A. M., Vasic V. (2013). Acetylcholinesterase
inhibitors: pharmacology and toxicology. Curr.
Neuropharmacol..

[57] Atanasova M., Stavrakov G., Philipova L., Zheleva D., Yordanov N., Doytchinova I. (2015). Galantamine
derivatives with indole moiety: docking,
design, synthesis and acetylcholinesterase inhibitory activity. Bioorg. Med. Chem..

[58] Wu M. Y., Esteban G., Brogi S., Shionoya M., Wang I., Campiani G., Unzeta M., Inokuchi T., Butini S., Marco-Contelles J. (2016). Donepezil-like
multifunctional agents: design, synthesis,
molecular modeling and biological evaluation. Eur. J. Med. Chem..

[59] Genest D., Rochais C., Lecoutey C., Oliveira Santos J. S. d., Ballandonne C., Butt-Gueulle S., Legay R., Since M., Dallemagne P. (2013). Design, synthesis
and biological evaluation of novel
indano-and thiaindano-pyrazoles with potential interest for Alzheimer’s
disease. Med. Chem. Commun..

[60] Karaburun A. C. ¸., Kaya Çavuşoğlu B., Acar Çevik U., Osmaniye D., Sağlık B.
N., Levent S., Özkay Y., Atlı O. ¨., Koparal A. S., Kaplancıklı Z. A. (2019). Synthesis
and antifungal potential of some novel benzimidazole-1, 3, 4-oxadiazole
compounds. Molecules.

[61] Acar
Cevik U., Saglik B. N., Levent S., Osmaniye D., Kaya Cavuşoglu B., Ozkay Y., Kaplancikli Z. A. (2019). Synthesis
and AChE-inhibitory activity of new benzimidazole derivatives. Molecules.

[62] Schrödinger Release 2021–3: LigPrep. Schrödinger, LLC, (NY): New York; 2021.

[63] Şahin I. ˙., Çeşme M., Özgeriş F. B., Tümer F. (2023). Triazole based
novel molecules as potential therapeutic
agents: Synthesis, characterization, biological evaluation, in-silico
ADME profiling and molecular docking studies. Chem. Biol. Interact..

[64] Kelder J., Grootenhuis P. D., Bayada D. M., Delbressine L. P., Ploemen J. P. (1999). Polar molecular
surface as a dominating determinant
for oral absorption and brain penetration of drugs. Pharm. Res..

[65] Kaya B., Tahtacı H., Çiftçi B., Duran H. E., Necip A., Işık M., Beydemir S. ¸. (2025). Discovery
of hydrazine clubbed thiazoles as potential antidiabetic agents: synthesis,
biological evaluation, and molecular docking studies. Drug Dev. Res..

[66] Clark D. E. (1999). Rapid calculation
of polar molecular surface area and its application to the prediction
of transport phenomena. 1. Prediction of intestinal absorption. J. Pharm. Sci..

[67] Lipinski C. A., Lombardo F., Dominy B. W., Feeney P. J. (1997). Experimental and
computational approaches to estimate solubility and permeability in
drug discovery and development settings. Adv.
Drug Delivery Rev..

[68] Al
Ati G., Chkirate K., El-Guourrami O., Chakchak H., Tüzün B., Mague J. T., Benzeid H., Achour R., Essassi E. M. (2024). Schiff
base compounds constructed from pyrazole–acetamide: Synthesis,
spectroscopic characterization, crystal structure, DFT, molecular
docking and antioxidant activity. J. Mol. Struct..

[69] Veber D. F., Johnson S. R., Cheng H. Y., Smith B. R., Ward K. W., Kopple K. D. (2002). Molecular properties
that influence the oral bioavailability
of drug candidates. J. Med. Chem..

[70] Pajouhesh H., Lenz G. R. (2005). Medicinal chemical properties of successful central
nervous system drugs. NeuroRx.

[71] Guengerich F. P. (2008). Cytochrome
p450 and chemical toxicology. Chem. Res. Toxicol..

[72] Martin Y. C. (2005). A bioavailability
score. J. Med. Chem..

[73] Bostancı H. E., Çevik U. A., Kapavarapu R., Güldiken Y. C., Inan Z. S. ¸., Güler O. ¨.
O. ¨., Uysal T. K., Uytun A., Çetin F. N., Özkay Y., Kaplancıklı Z. A. (2023). Synthesis, biological
evaluation and in silico studies of novel thiadiazole-hydrazone derivatives
for carbonic anhydrase inhibitory and anticancer activities. SAR QSAR Environ. Res..

[74] Ünver H., Acar Cevik U., Bostancı H. E., Kaya O., Kocyigit Ü. M. (2023). Imidazole-hydrazone
derivatives: Synthesis, characterization, X-ray
structures and evaluation of anticancer and carbonic anhydrase I–II
inhibition properties. ChemistrySelect.

[75] Işık A., Acar Çevik U., Karayel A., Ahmad I., Patel H., Çelik İ., Gül Ü.
D., Bayazıt G., Bostancı H. E., Koçak A., Özkay Y., Kaplancıklı Z. A. (2024). Synthesis, DFT calculations, in silico
studies, and antimicrobial evaluation of benzimidazole-thiadiazole
derivatives. ACS Omega.

[76] Faris M., Bostancı H. E., Özcan I., Öztürk M., Koçyiğit U. M., Erdoğan T., Tahtaci H. (2024). Imidazole-derived alkyl and aryl ethers: synthesis,
characterization, in vitro anticancer and antioxidant activities,
carbonic anhydrase I–II inhibition properties, and in silico
studies. ACS Omega.

[77] Acar
Çevik U., Osmaniye D., Sağlik B. N., Levent S., Kaya Çavuşoğlu B., Özkay Y., Kaplancikli Z. A. (2019). Synthesis and evaluation of new pyrazoline-thiazole
derivatives as monoamine oxidase inhibitors. J. Heterocycl. Chem..

[78] Ellman G.
L., Courtney K. D., Andres V., Feather-Stone R. M. (1961). A new and
rapid colorimetric determination of acetylcholinesterase activity. Biochem. Pharmacol..

[79] Gülçin I. ˙., Scozzafava A., Supuran C. T., Akıncıoğlu H., Koksal Z., Turkan F., Alwasel S. (2016). The effect of caffeic
acid phenethyl ester (CAPE) on metabolic enzymes including acetylcholinesterase,
butyrylcholinesterase, glutathione S-transferase, lactoperoxidase,
and carbonic anhydrase isoenzymes I, II, IX, and XII. J. Enzyme Inhib. Med. Chem..

[80] Lineweaver H., Burk D. (1934). The determination of enzyme dissociation
constants. J. Am. Chem. Soc..

[81] Demir Y., Işık M., Gülçin I. ˙., Beydemir S. ¸. (2017). Phenolic compounds inhibit the aldose reductase enzyme
from the sheep kidney. J. Biochem. Mol. Toxicol..

[82] Cheung J., Rudolph M. J., Burshteyn F., Cassidy M. S., Gary E. N., Love J., Franklin M. C., Height J. J. (2012). Structures of human
acetylcholinesterase in complex with pharmacologically important ligands. J. Med. Chem..

[83] Nicolet Y., Lockridge O., Masson P., Fontecilla-Camps J. C., Nachon F. (2013). Crystal structure of
human butyrylcholinesterase and
of its complexes with substrate and products. J. Biol. Chem..

[84] Sussman J. L., Harel M., Frolow F., Oefner C., Goldman A., Toker L., Silman I. (1991). Atomic structure
of acetylcholinesterase
from Torpedo californica: a prototypic acetylcholine-binding protein. Science.

[85] Dvir H., Silman I., Harel M., Rosenberry T. L., Sussman J. L. (2010). Acetylcholinesterase: from 3D structure
to function. Chem. Biol. Interact..

[86] Schrödinger Release 2021–3: Maestro. Schrödinger, LLC, (NY): New York; 2021.

[87] Singh A., Kaur H., Singh H., Singh B., Bedi P. M. S., Kaur S. (2023). Ameliorative effects of Grewia asiatica leaves in animal
models of pain and inflammation. J. Herbs Spices
Med. Plants.

[88] Shahzadi I., Zahoor A. F., Tüzün B., Mansha A., Anjum M. N., Rasul A., Irfan A., Kotwica-Mojzych K., Mojzych M. (2022). Repositioning of acefylline as anti-cancer
drug: Synthesis,
anticancer and computational studies of azomethines derived from acefylline
tethered 4-amino-3-mercapto-1, 2, 4-triazole. PLoS One.

[89] Schrödinger Release 2021–3: QikProp. Schrödinger, LLC, (NY): New York; 2021.

