# Supplementary material for: Design, Synthesis, and Molecular Docking Studies of Novel Pyrazoline-Thiazoles as Cholinesterase Dual-Target Inhibitors for the Treatment of Alzheimer’s Disease
Source: ACS Omega. 2025 Aug 22;10(34):38427–39. doi: 10.1021/acsomega.5c01055 (PMC12409578; doi:10.1021/acsomega.5c01055)
Supplement: Supplementary file 1 [file ao5c01055_si_001.pdf]

Design, Synthesis and Molecular Docking Studies of Novel Pyrazoline-Thiazoles as Cholinesterase Dual-Target Inhibitors for the Treatment of Alzheimer's Disease

Betül Kaya[1], Ulviye Acar Çevik[2,3], Bilge Çiftçi[4], Adem Necip[5], Mesut IŞIK[6], Ebru Nur Ay[7], Süleyman Yur[3], Yusuf Özkay[2], Şükrü Beydemir[8], Zafer Asım Kaplancıklı[2,9]

[1]Vocational School of Health Services, Pharmacy Services, Bilecik Şeyh Edebali University, 11230, Bilecik, Turkey.

[2]Department of Pharmaceutical Chemistry, Faculty of Pharmacy, Anadolu University, 26470 Eskişehir, Turkey.

[3]Medicinal Plant, Drug and Scientific Research and Application Center (AUBIBAM), Anadolu University, Eskisehir, 26470 Turkey.

[4]Vocational School of Health Services, Bilecik Şeyh Edebali University, 11230, Bilecik, Turkey

[5]Department of Pharmacy Services, Vocational School of Health Services, Harran University, 63300, Şanlıurfa, Turkey.

[6]Department of Bioengineering, Faculty of Engineering, Bilecik Şeyh Edebali University, 11230, Bilecik, Turkey.

[7]Department of Molecular Biology and Genetics, Faculty of Engineering and Natural Sciences, İstinye University, Turkey.

[8]Department of Biochemistry, Faculty of Pharmacy, Anadolu University, 26470, Eskişehir, Turkey.

[9]The Rectorate of Bilecik Şeyh Edebali University, 11230, Bilecik, Turkey.

*Corresponding Author. E-mail: zakaplan@anadolu.edu.tr

Address:

Department of Pharmaceutical Chemistry, Faculty of Pharmacy, Anadolu University, 26470 Eskişehir, Turkey.

The Rectorate of Bilecik Şeyh Edebali University, 11230, Bilecik, Turkey.

Data File C:\Chem32\1\Data\BNJ 2025-06-13 12-55-54\001-P1-A1-BNJ1.D
Sample Name: BNJ1

===

Acq. Operator : SYSTEM Seq. Line : 1
Acq. Instrument : HPLC DAD Location : P1-A1
Injection Date : 6/13/2025 12:56:46 PM Inj : 1
Inj Volume : 2.000 µl
Acq. Method : C:\Chem32\1\Data\BNJ 2025-06-13 12-55-54\DENEME.M
Last changed : 6/13/2025 12:55:54 PM by SYSTEM
Analysis Method : C:\Chem32\1\Data\BNJ 2025-06-13 12-55-54\DENEME.M (Sequence Method)
Last changed : 6/24/2025 11:40:09 AM by SYSTEM
(modified after loading) (Current integration events modified)

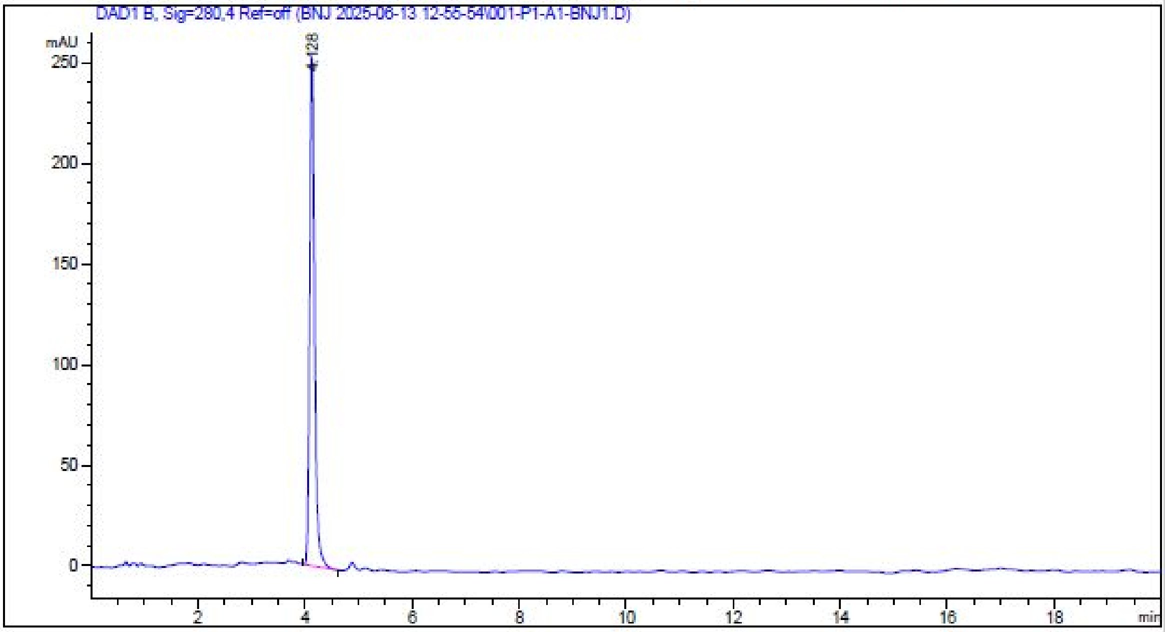

===

Area Percent Report

===

Sorted By : Signal
Multiplier : 1.0000
Dilution : 1.0000
Do not use Multiplier & Dilution Factor with ISTDs

Signal 1: DAD1 B, Sig=280,4 Ref=off

Peak #	RetTime [min]	Type	Width [min]	Area [mAU*s]	Height [mAU]	Area %
1	4.128	BB	0.0981	1604.87708	252.70345	100.0000

Totals : 1604.87708 252.70345

Figure S1. HPLC chromatogram of compound **3a**

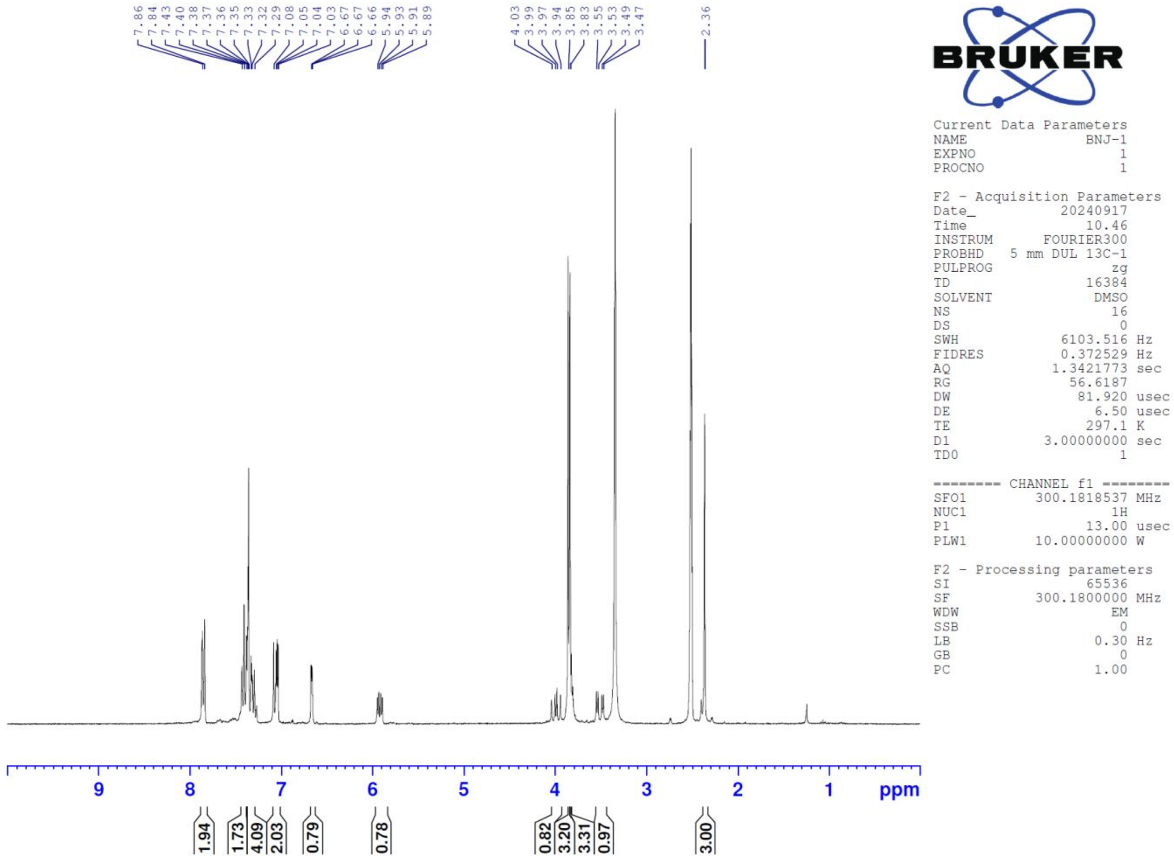

Figure S2. ^{1}H-NMR spectrum of compound **3a**

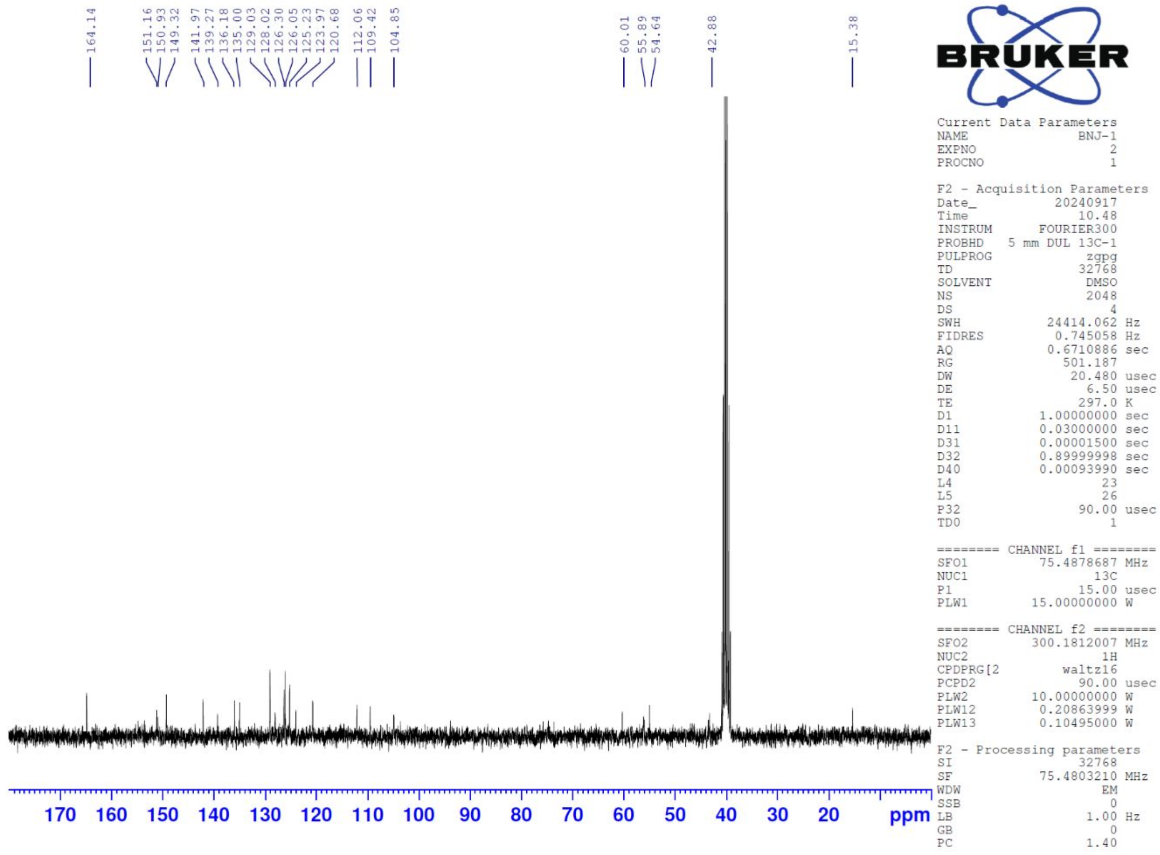

Figure S3. ^{13}C-NMR spectrum of compound **3a**

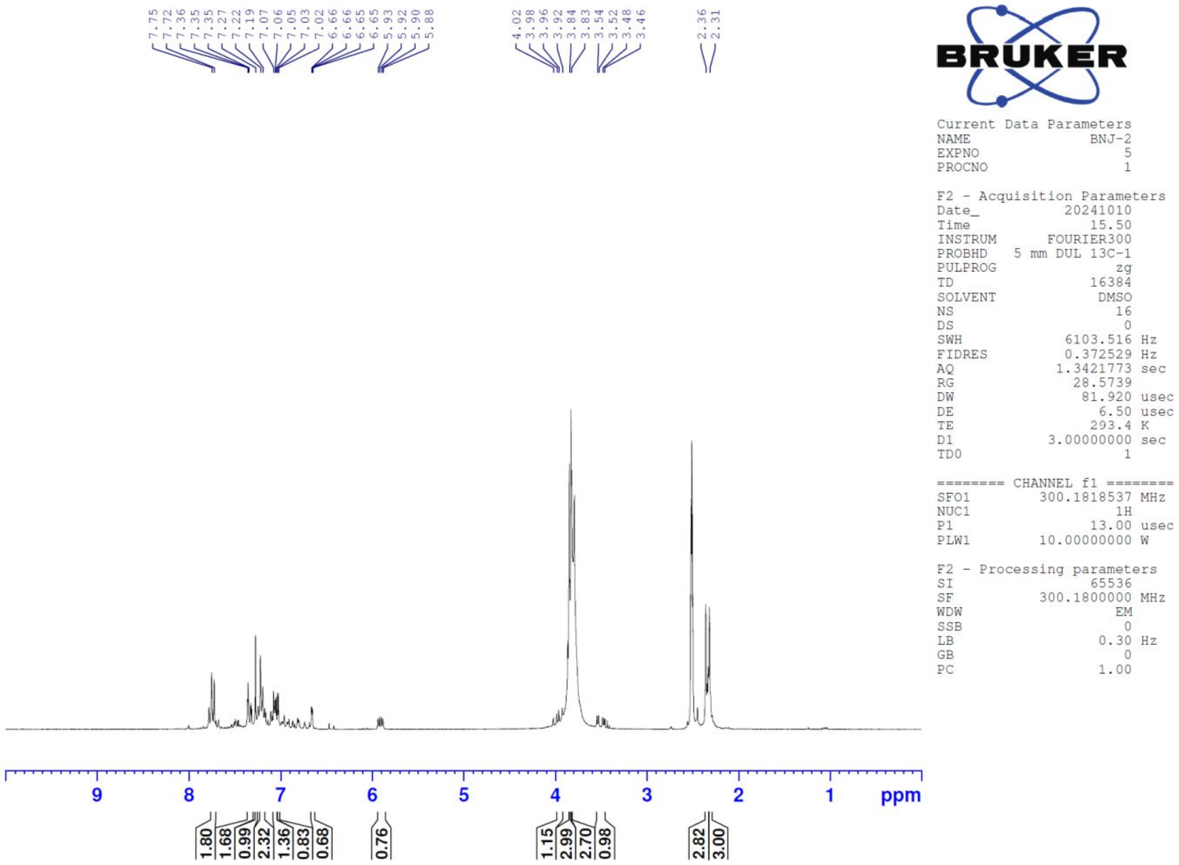

Figure S4. ^{1}H-NMR spectrum of compound **3b**

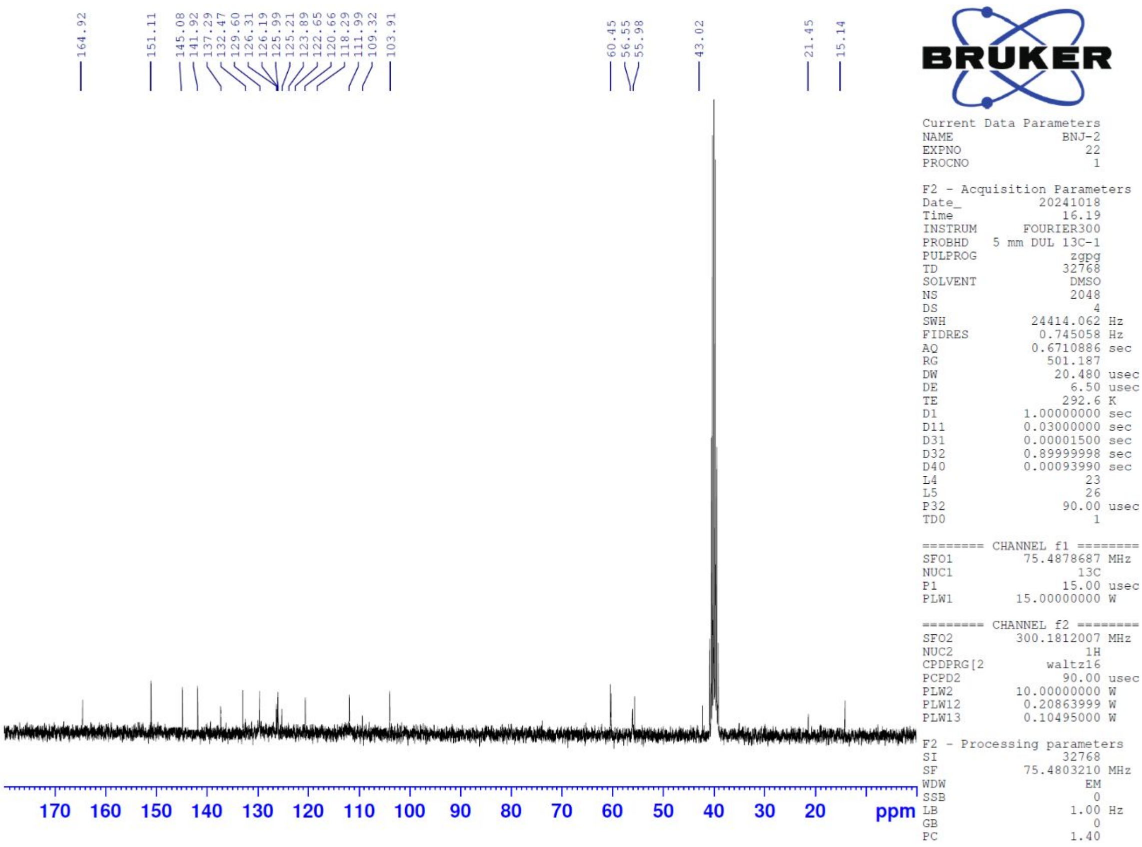

Figure S5. ^{13}C-NMR spectrum of compound **3b**

Data File C:\Chem32\1\Data\BNJ 2025-06-13 12-55-54\005-P1-A5-BNJ3.D
Sample Name: BNJ3

===

Acq. Operator : SYSTEM Seq. Line : 5
Acq. Instrument : HPLC DAD Location : P1-A5
Injection Date : 6/13/2025 2:20:19 PM Inj : 1
Inj Volume : 2.000 µl
Acq. Method : C:\Chem32\1\Data\BNJ 2025-06-13 12-55-54\DENEME.M
Last changed : 6/13/2025 12:55:54 PM by SYSTEM
Analysis Method : C:\Chem32\1\Data\BNJ 2025-06-13 12-55-54\DENEME.M (Sequence Method)
Last changed : 6/24/2025 11:42:27 AM by SYSTEM
(modified after loading) (Current integration events modified)

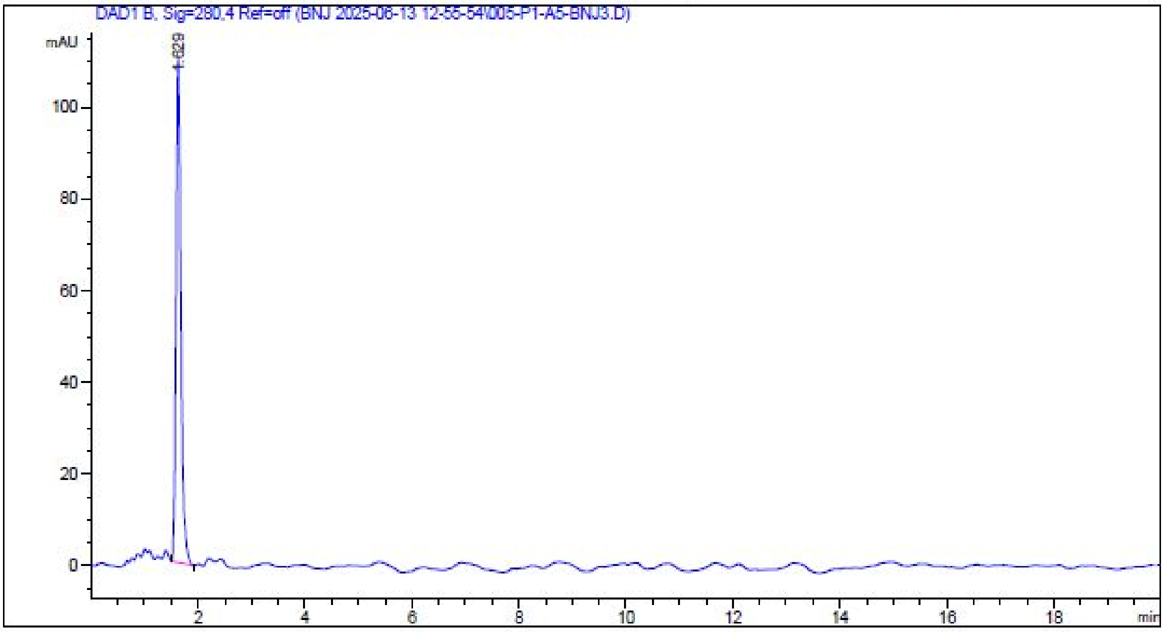

===

Area Percent Report

===

Sorted By : Signal
Multiplier : 1.0000
Dilution : 1.0000
Do not use Multiplier & Dilution Factor with ISTDs

Signal 1: DAD1 B, Sig=280,4 Ref=off

Peak #	RetTime [min]	Type	Width [min]	Area [mAU*s]	Height [mAU]	Area %
1	1.629	BB	0.0967	704.36542	110.06890	100.0000

Totals : 704.36542 110.06890

Figure S6. HPLC chromatogram of compound **3c**

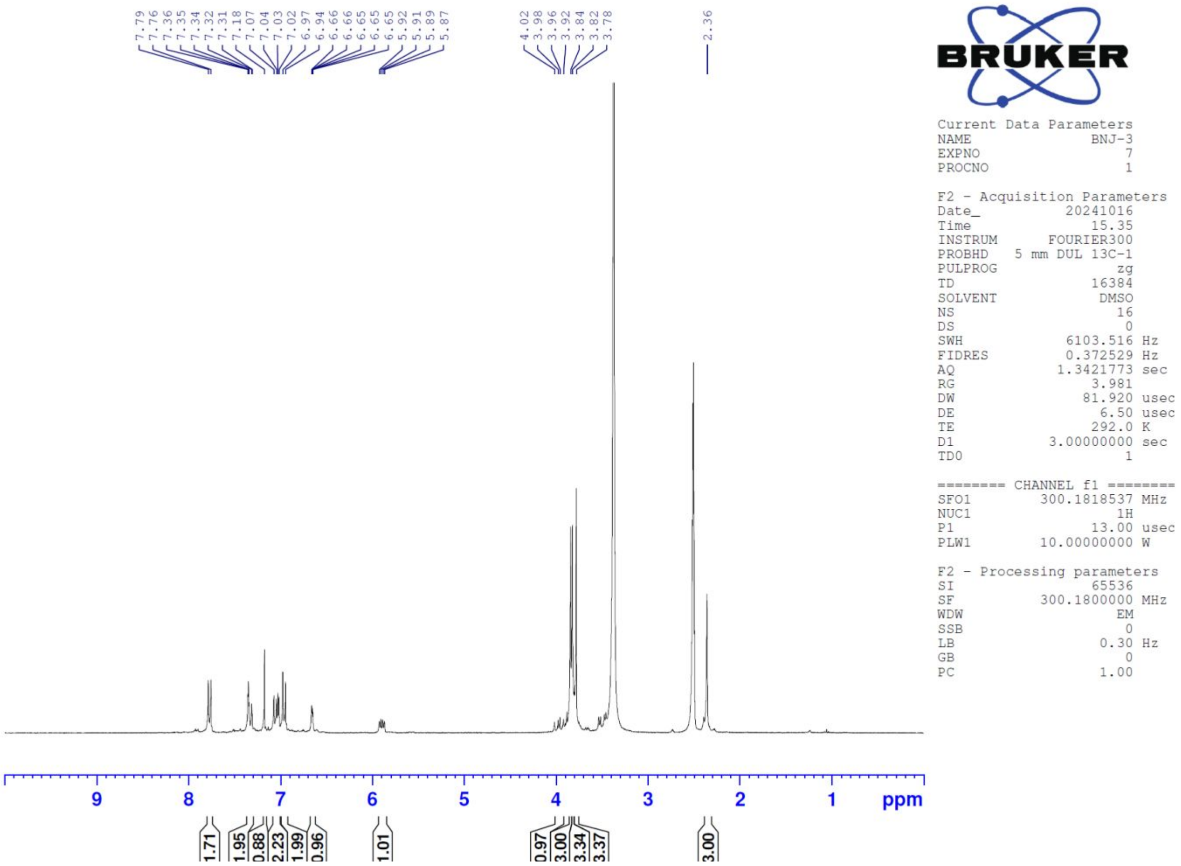

Figure S7. ^{1}H-NMR spectrum of compound **3c**

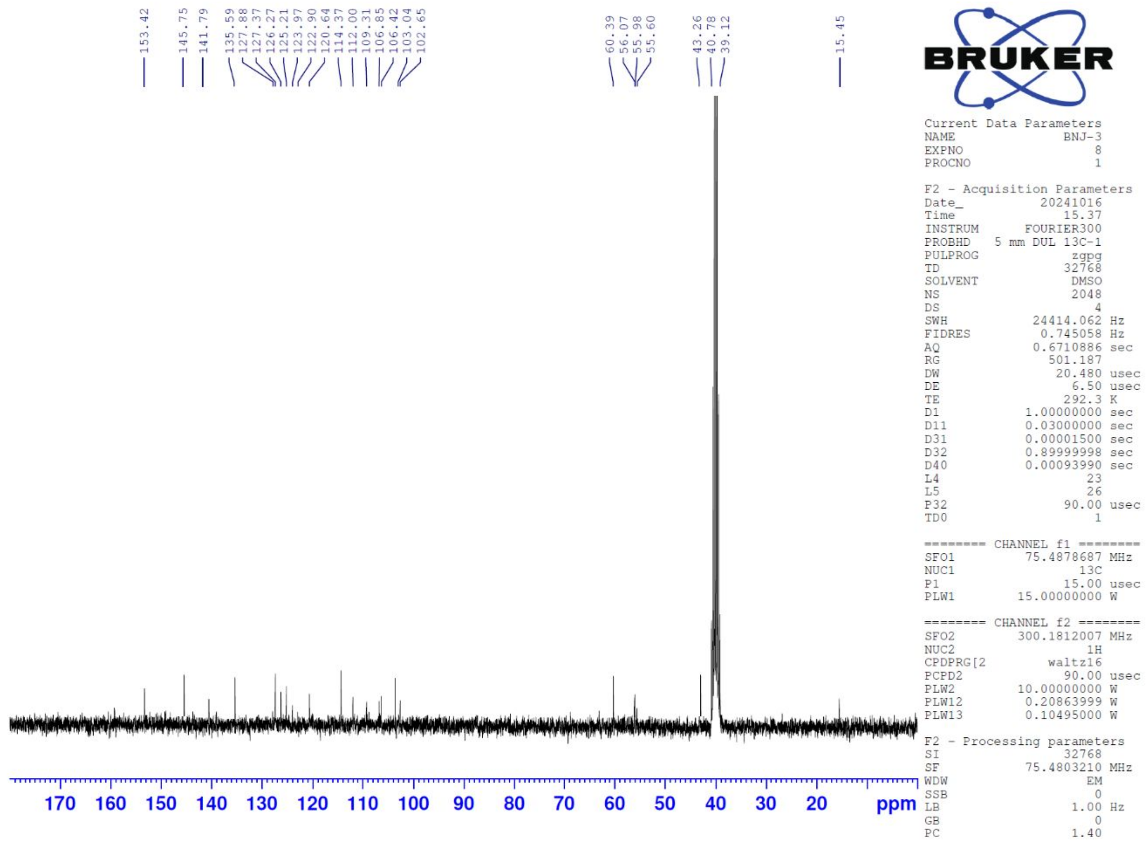

Figure S8. ^{13}C-NMR spectrum of compound **3c**

```
Data File C:\Chem32\1\Data\BNJ 2025-06-24 09-02-23\004-P1-B4-BNJ4_.D
Sample Name: BNJ4*

    =====================================================================
    Acq. Operator   : SYSTEM                          Seq. Line :   4
    Acq. Instrument : HPLC DAD                         Location :   P1-B4
    Injection Date  : 6/24/2025 10:05:57 AM                 Inj :   1
                                                    Inj Volume : 2.000 µl
    Acq. Method     : C:\Chem32\1\Data\BNJ 2025-06-24 09-02-23\Deneme.M
    Last changed    : 6/24/2025 9:02:23 AM by SYSTEM
    Analysis Method : C:\Chem32\1\Data\BNJ 2025-06-24 09-02-23\Deneme.M (Sequence Method)
    Last changed    : 6/24/2025 11:41:47 AM by SYSTEM
                      (modified after loading)  (Current integration events modified)
```

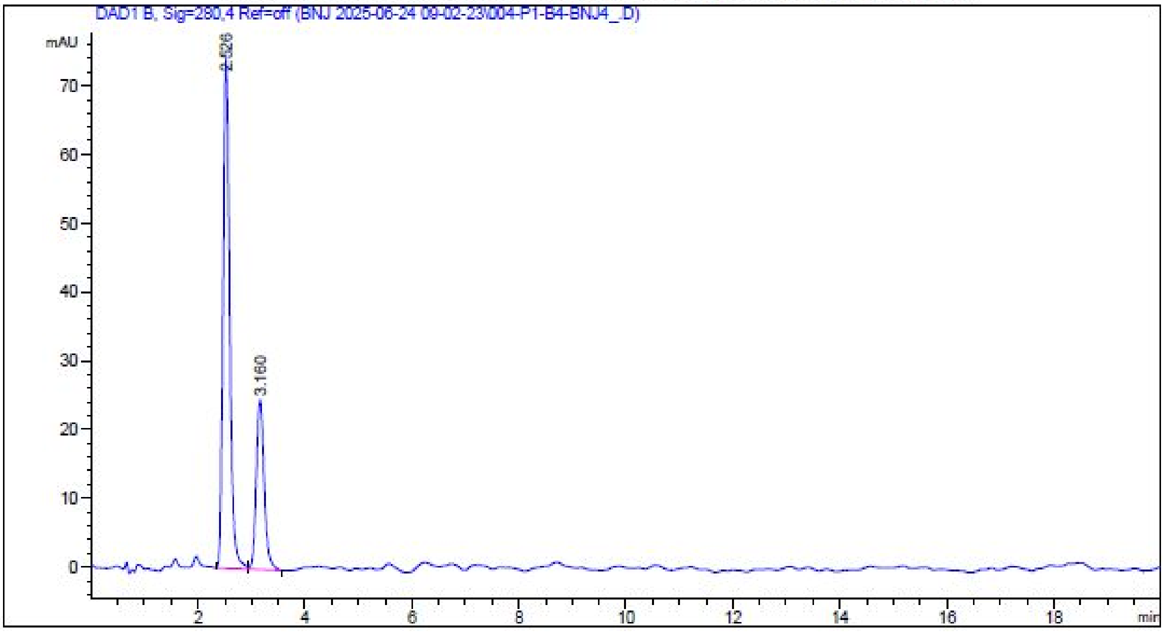


```
    =====================================================================
                             Area Percent Report
    =====================================================================

    Sorted By             :      Signal
    Multiplier            :      1.0000
    Dilution              :      1.0000
    Do not use Multiplier & Dilution Factor with ISTDs


    Signal 1: DAD1 B, Sig=280,4 Ref=off

    Peak RetTime Type  Width     Area      Height     Area
      #   [min]        [min]   [mAU*s]     [mAU]        %
    ----|-------|----|-------|----------|----------|--------|
       1   2.526 BV    0.1323  638.73450   74.26569  71.5215
       2   3.160 VB    0.1580  254.33232   24.73545  28.4785

    Totals :                   893.06682   99.00114
```

Figure S9. HPLC chromatogram of compound **3d**

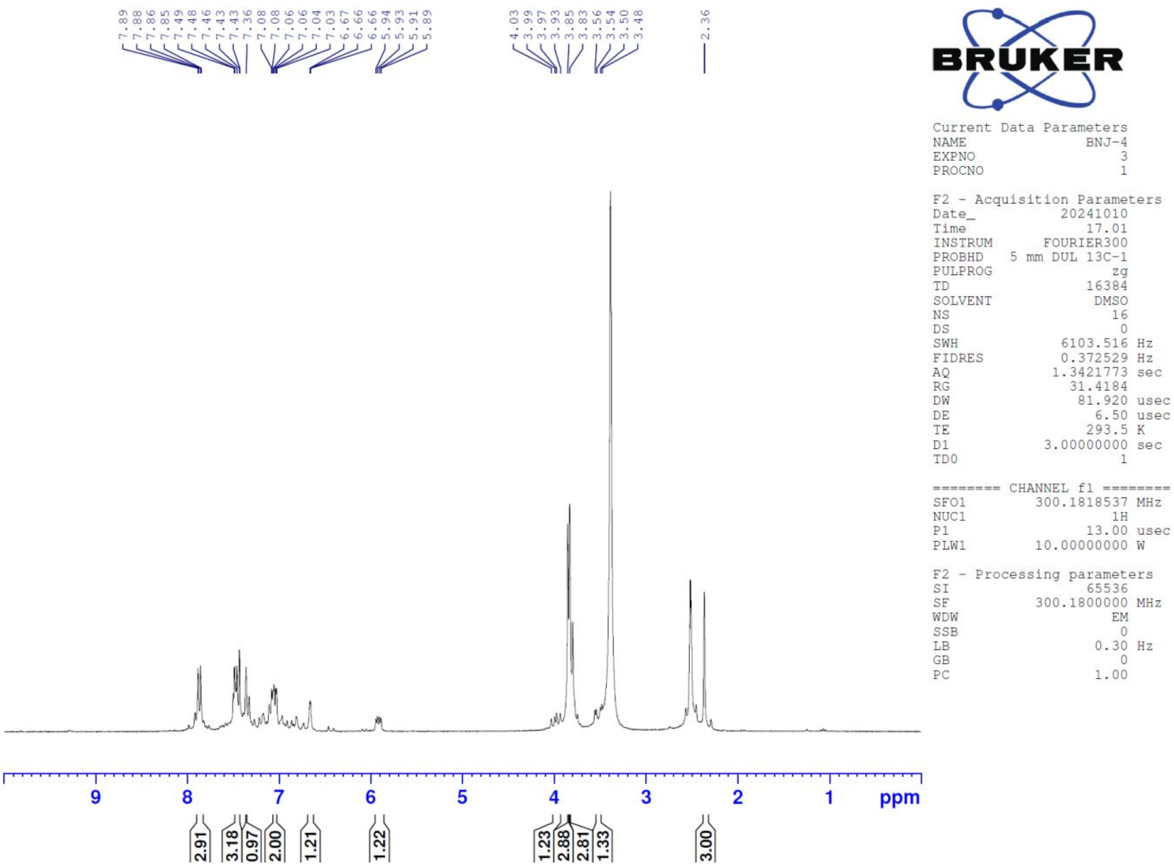

Figure S10. ^{1}H-NMR spectrum of compound **3d**

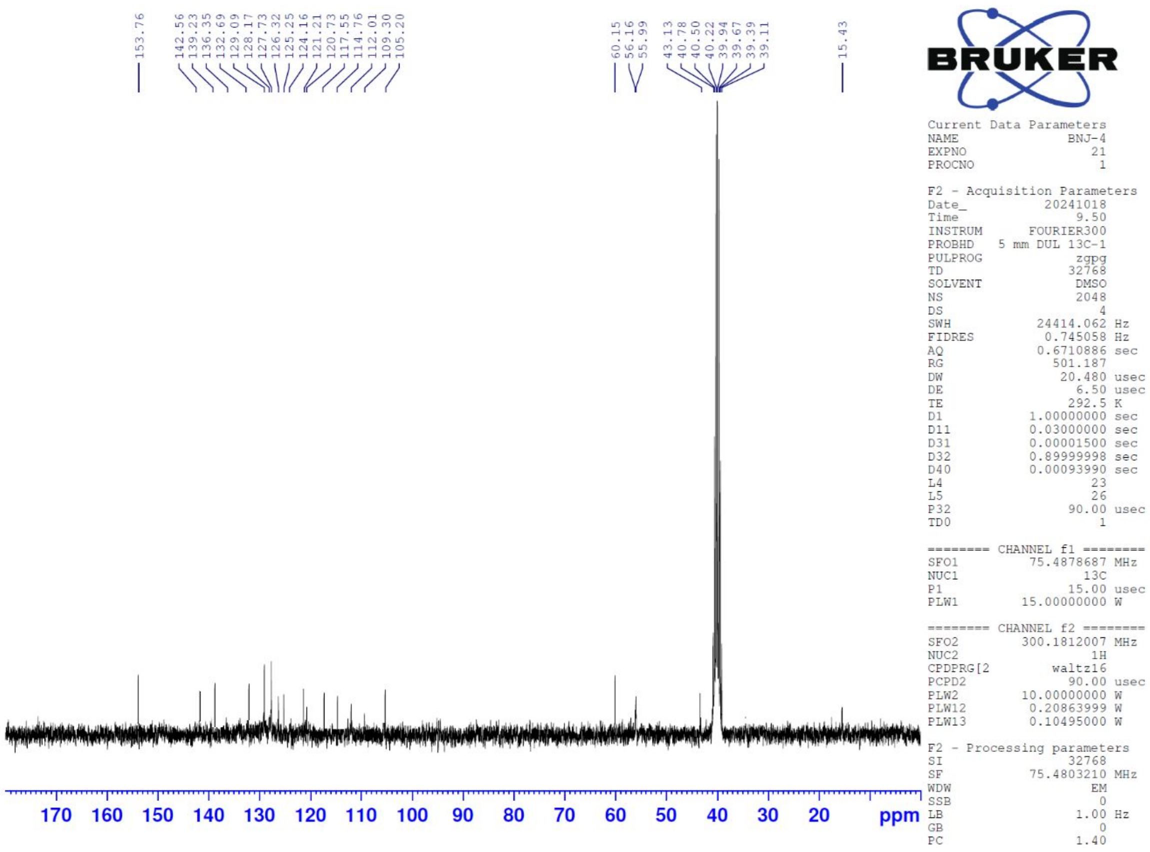

Figure S11. ^{13}C-NMR spectrum of compound **3d**

Data File C:\Chem32\1\Data\BNJ 2025-06-24 09-02-23\005-P1-B5-BNJ5_.D
Sample Name: BNJ5*

===

Acq. Operator	: SYSTEM	Seq. Line :	5
Acq. Instrument	: HPLC DAD	Location :	P1-B5
Injection Date	: 6/24/2025 10:26:51 AM	Inj :	1
		Inj Volume :	2.000 µl

Acq. Method : C:\Chem32\1\Data\BNJ 2025-06-24 09-02-23\Deneme.M
Last changed : 6/24/2025 9:02:23 AM by SYSTEM
Analysis Method : C:\Chem32\1\Data\BNJ 2025-06-24 09-02-23\Deneme.M (Sequence Method)
Last changed : 6/24/2025 11:41:52 AM by SYSTEM
(modified after loading) (Current integration events modified)

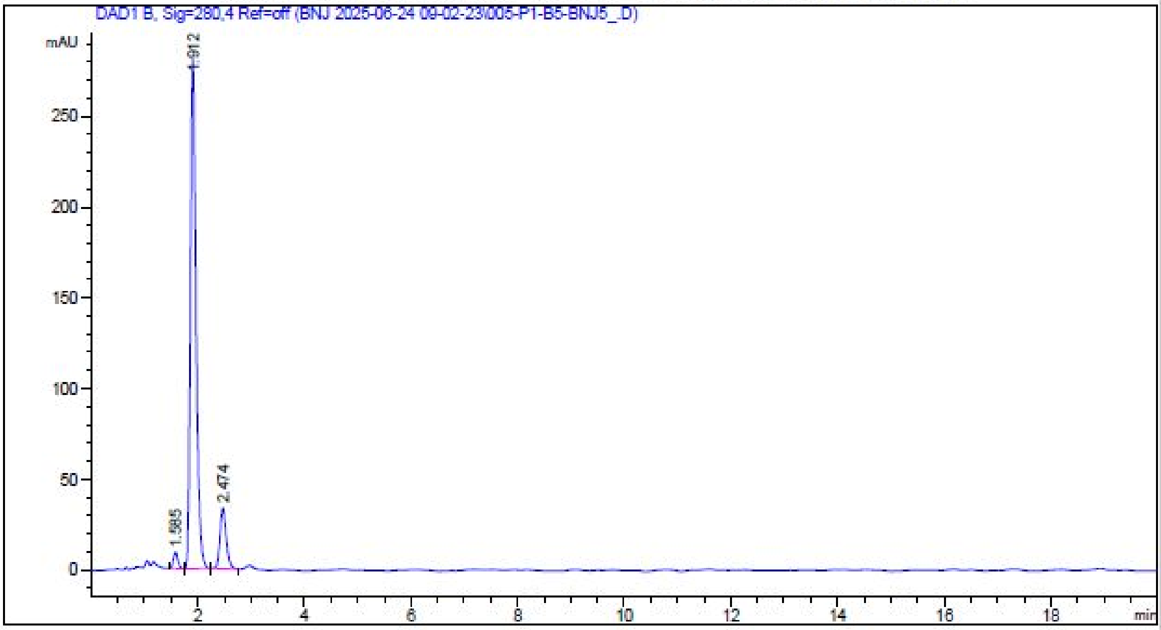

===
Area Percent Report
===

Sorted By : Signal
Multiplier : 1.0000
Dilution : 1.0000
Do not use Multiplier & Dilution Factor with ISTDs

Signal 1: DAD1 B, Sig=280,4 Ref=off

Peak #	RetTime [min]	Type	Width [min]	Area [mAU*s]	Height [mAU]	Area %
1	1.585	BB	0.0901	53.06063	9.09416	2.2317
2	1.912	BB	0.1107	2047.74426	282.06662	86.1279
3	2.474	BB	0.1266	276.75647	33.44188	11.6404
Totals :				2377.56137	324.60266	

Figure S12. HPLC chromatogram of compound **3e**

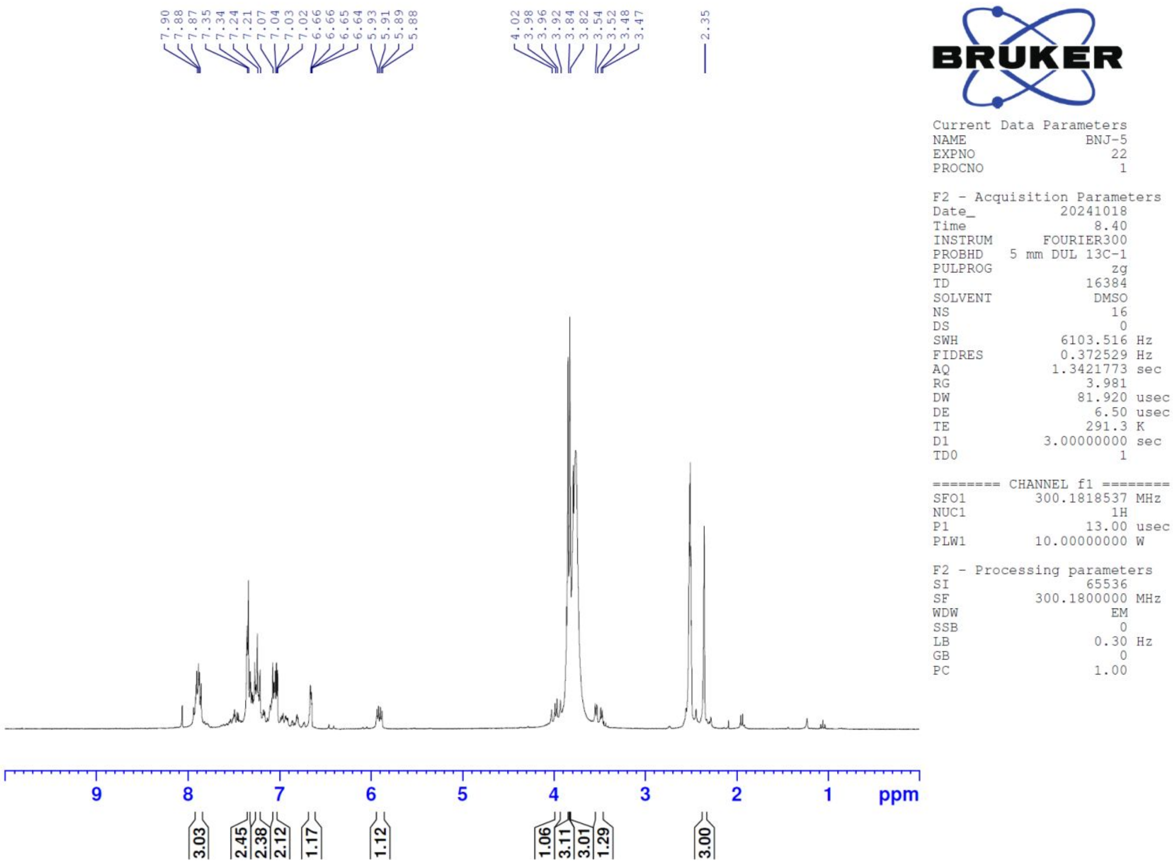

Figure S13. ^{1}H-NMR spectrum of compound **3e**

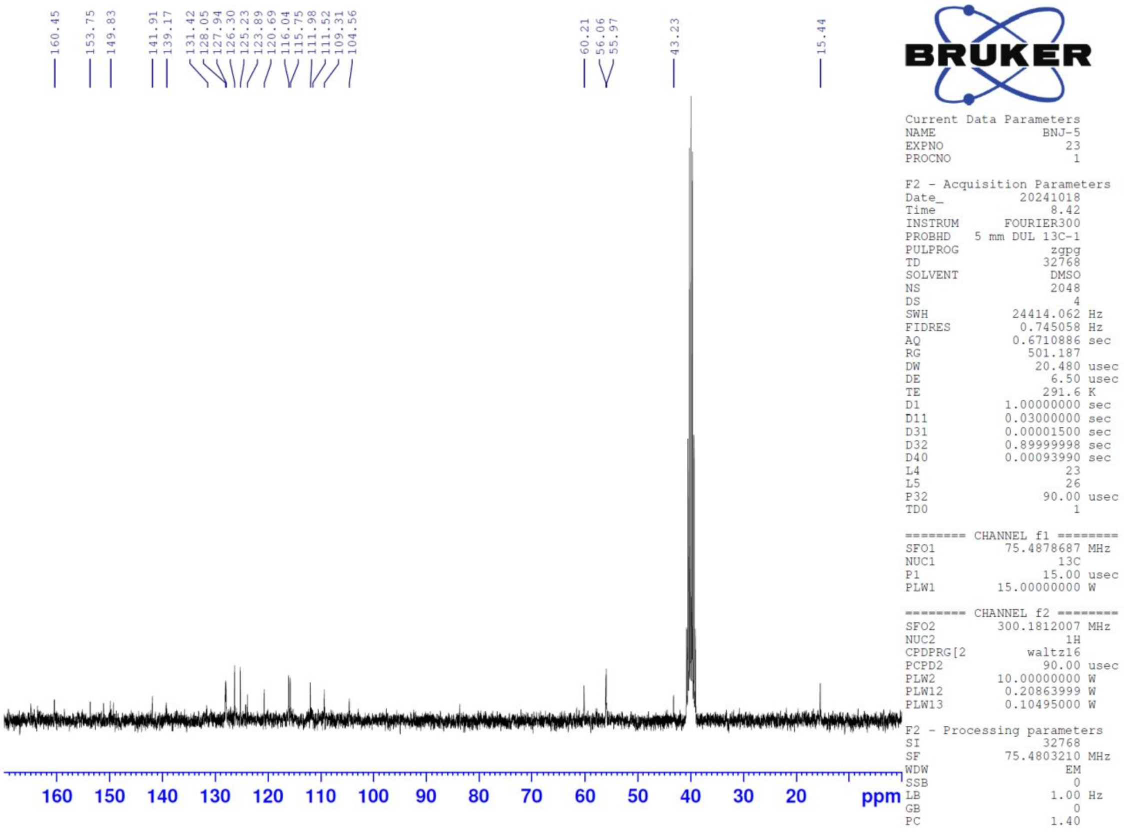

Figure S14. ^{13}C-NMR spectrum of compound **3e**

```
Data File C:\Chem32\1\Data\BNJ 2025-06-13 12-55-54\009-P1-A9-BNJ6.D
Sample Name: BNJ6

    =====================================================================
    Acq. Operator   : SYSTEM                          Seq. Line :   9
    Acq. Instrument : HPLC DAD                         Location :   P1-A9
    Injection Date  : 6/13/2025 3:43:49 PM                  Inj :   1
                                                    Inj Volume : 2.000 µl
    Acq. Method     : C:\Chem32\1\Data\BNJ 2025-06-13 12-55-54\DENEME.M
    Last changed    : 6/13/2025 12:55:54 PM by SYSTEM
    Analysis Method : C:\Chem32\1\Data\BNJ 2025-06-13 12-55-54\DENEME.M (Sequence Method)
    Last changed    : 6/24/2025 11:42:48 AM by SYSTEM
                      (modified after loading) (Current integration events modified)
```

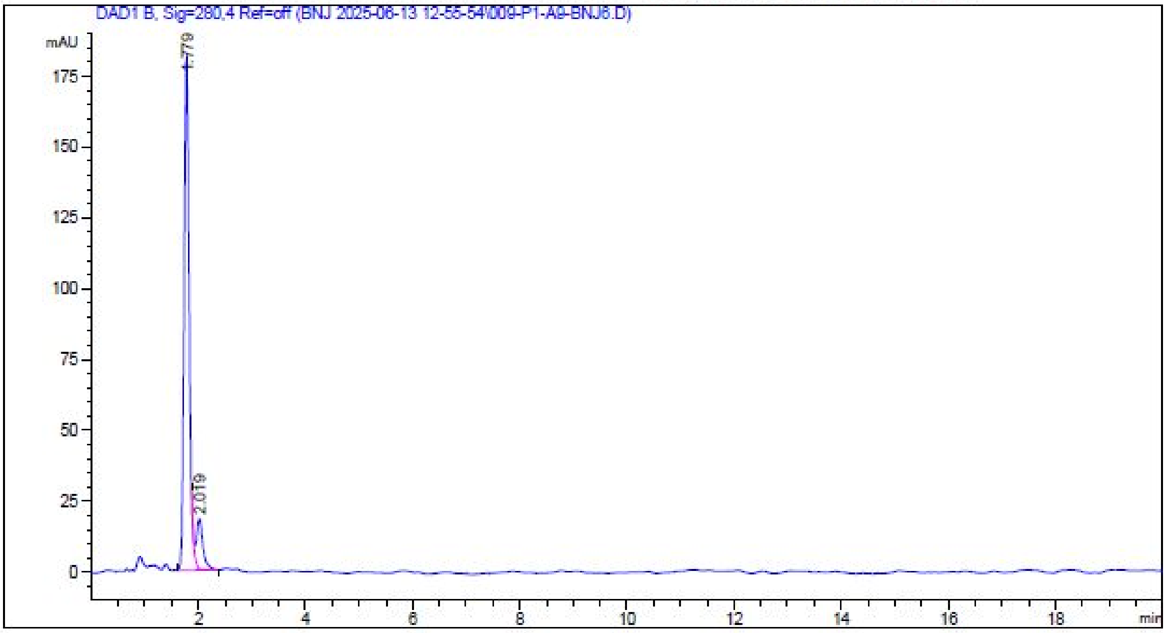


```
    =====================================================================
                              Area Percent Report
    =====================================================================

    Sorted By             :      Signal
    Multiplier            :      1.0000
    Dilution              :      1.0000
    Do not use Multiplier & Dilution Factor with ISTDs


    Signal 1: DAD1 B, Sig=280,4 Ref=off
```

Peak #	RetTime [min]	Type	Width [min]	Area [mAU*s]	Height [mAU]	Area %
1	1.779	BV R	0.1025	1218.14636	180.97578	89.3743
2	2.019	VB E	0.1220	144.82593	17.60763	10.6257

Totals : 1362.97229 198.58341

Figure S15. HPLC chromatogram of compound **3f**

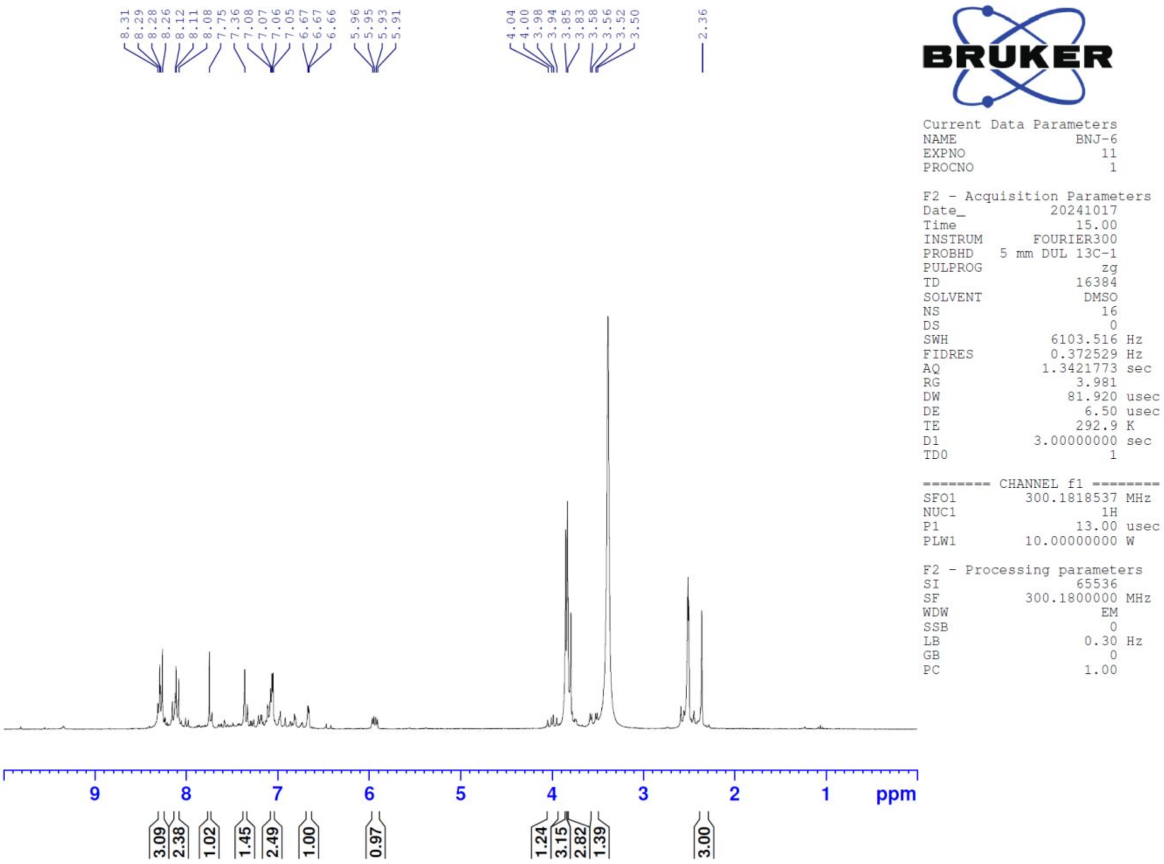

Figure S16. ^{1}H-NMR spectrum of compound **3f**

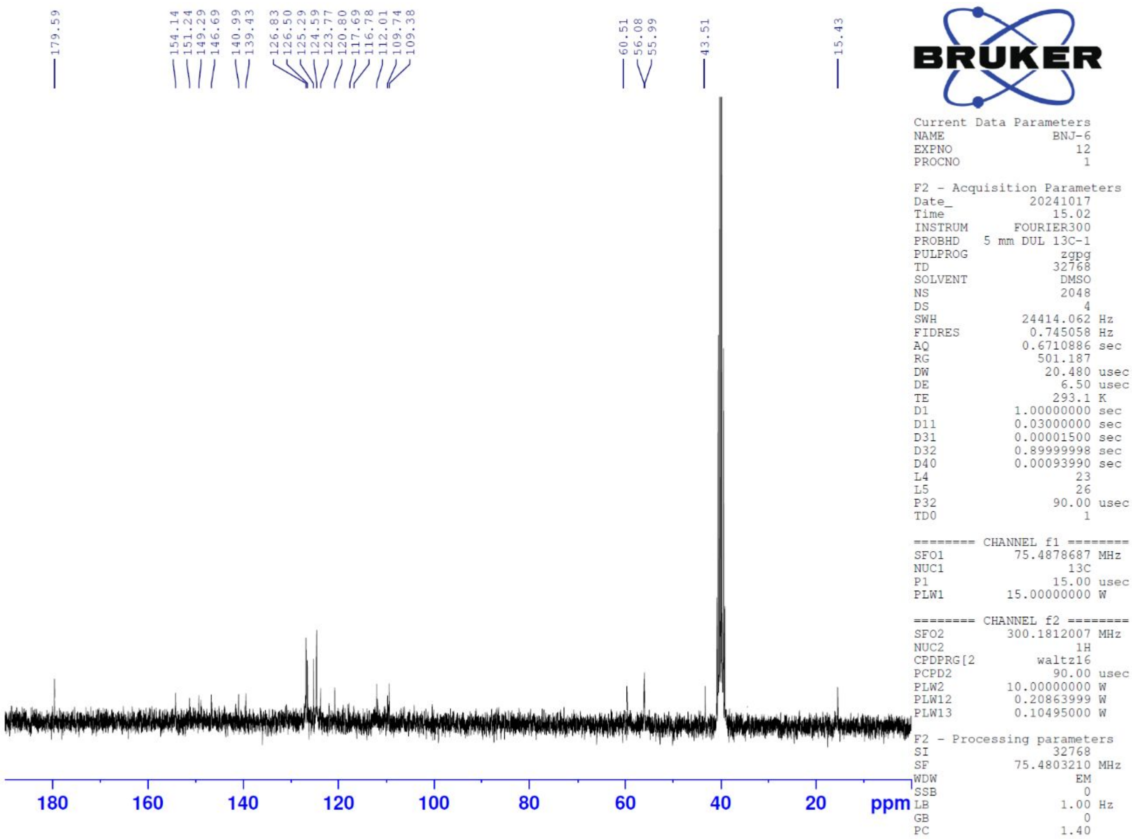

Figure S17. ^{13}C-NMR spectrum of compound **3f**

```
Data File C:\Chem32\1\Data\BNJ 2025-06-13 12-55-54\010-P1-A10-BNJ7.D
Sample Name: BNJ7

=====================================================================
Acq. Operator   : SYSTEM                        Seq. Line :  10
Acq. Instrument : HPLC DAD                       Location :   P1-A10
Injection Date  : 6/13/2025 4:04:42 PM                Inj :   1
                                               Inj Volume : 2.000 µl
Acq. Method     : C:\Chem32\1\Data\BNJ 2025-06-13 12-55-54\DENEME.M
Last changed    : 6/13/2025 12:55:54 PM by SYSTEM
Analysis Method : C:\Chem32\1\Data\BNJ 2025-06-13 12-55-54\DENEME.M (Sequence Method)
Last changed    : 6/24/2025 11:43:03 AM by SYSTEM
                  (modified after loading) (Current integration events modified)
```

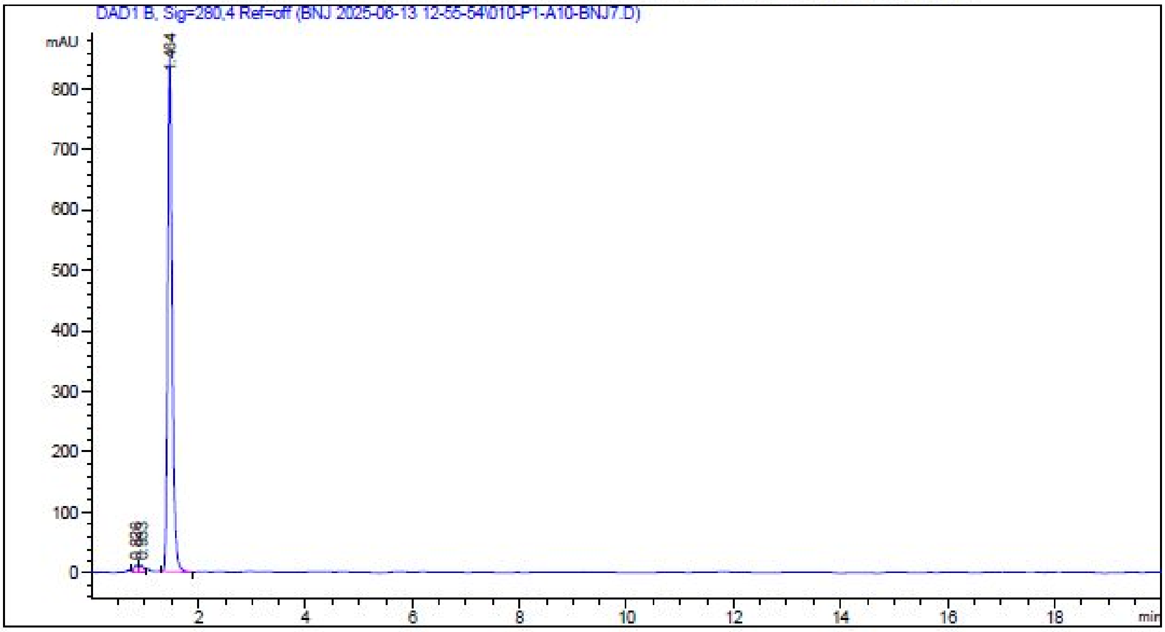


```
=====================================================================
                         Area Percent Report
=====================================================================

Sorted By             :      Signal
Multiplier            :      1.0000
Dilution              :      1.0000
Do not use Multiplier & Dilution Factor with ISTDs


Signal 1: DAD1 B, Sig=280,4 Ref=off
```

Peak #	RetTime [min]	Type	Width [min]	Area [mAU*s]	Height [mAU]	Area %
1	0.826	VV	0.0798	63.93494	11.68478	1.2343
2	0.933	VV	0.0769	56.73282	10.86952	1.0953
3	1.464	BB	0.0914	5059.12207	850.68036	97.6704
Totals :				5179.78983	873.23466	

Figure S18. HPLC chromatogram of compound **3g**

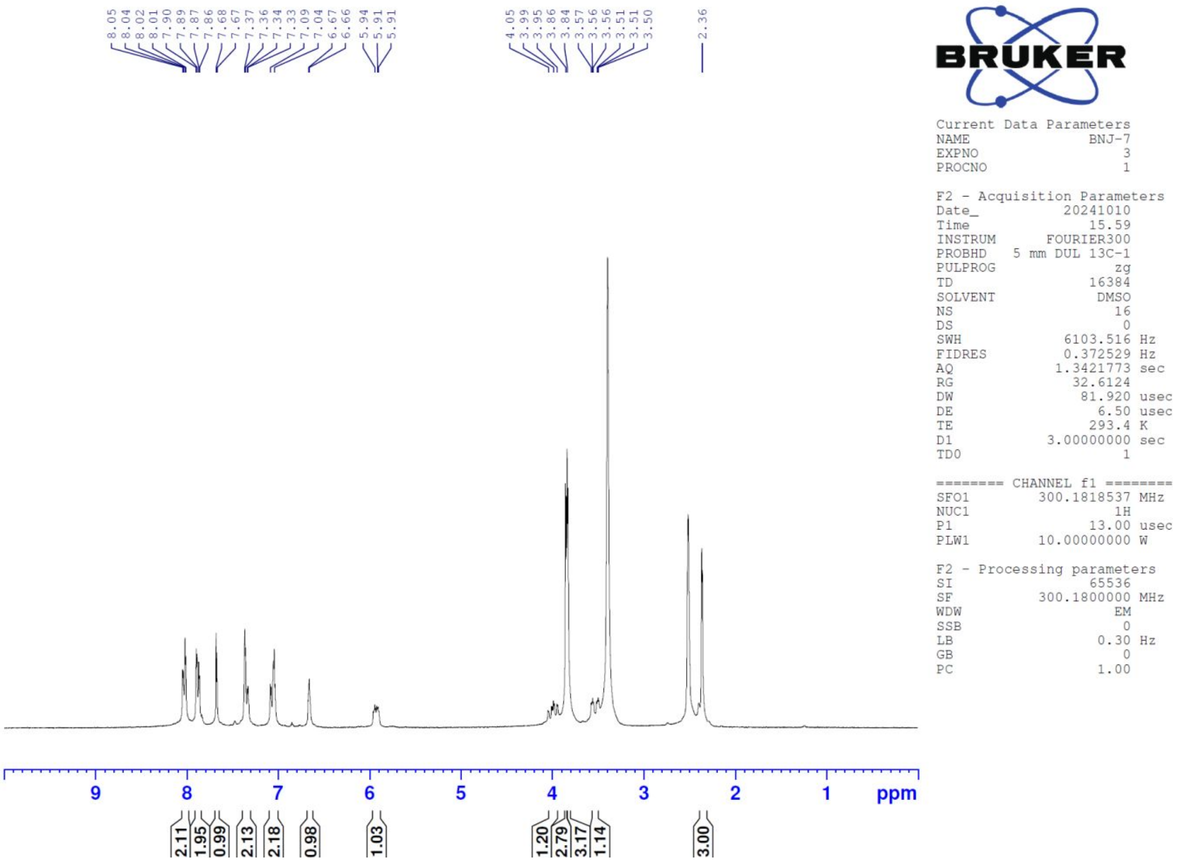

Figure S19. ^{1}H-NMR spectrum of compound **3g**

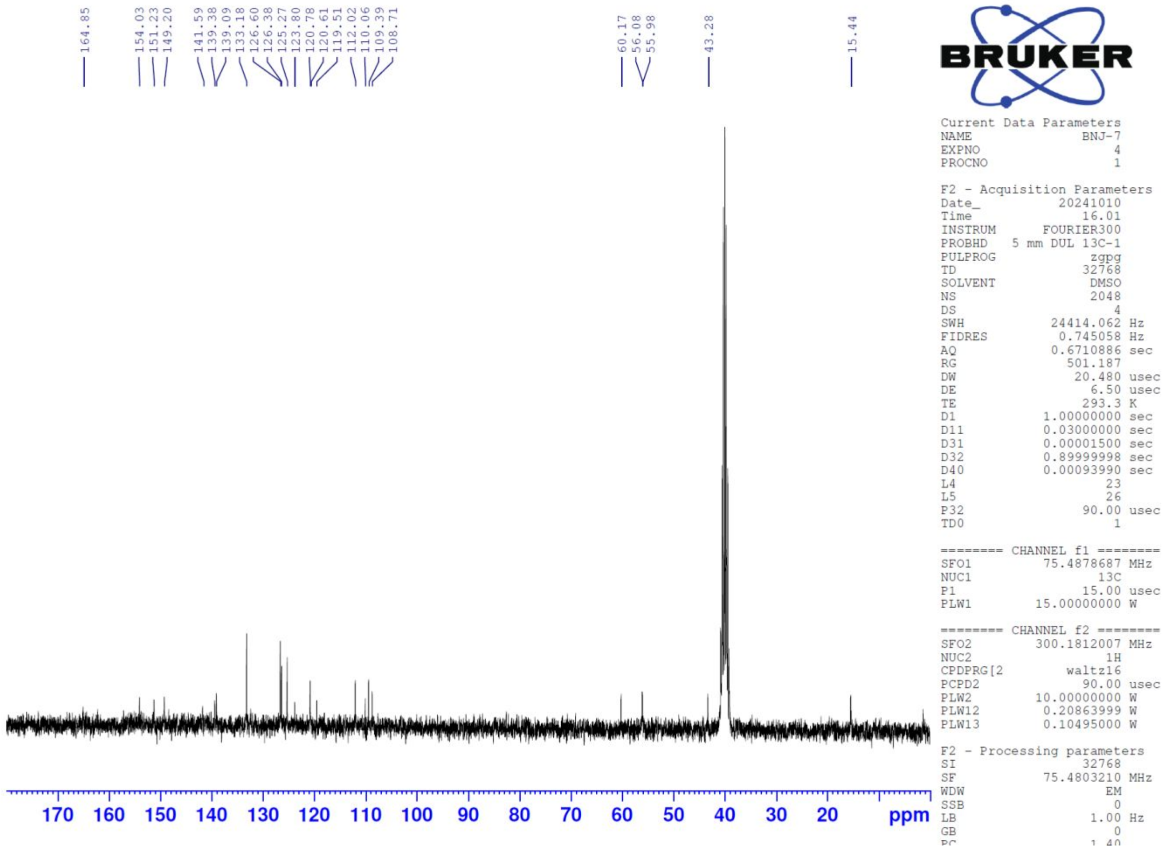

Figure S20. ^{13}C-NMR spectrum of compound **3g**

Data File C:\Chem32\1\Data\New 2025-06-12 14-20-20\001-P1-A1-BNJ8-4.D
Sample Name: BNJ8-4

===

Acq. Operator	: SYSTEM	Seq. Line :	1
Acq. Instrument	: HPLC DAD	Location :	P1-A1
Injection Date	: 6/12/2025 2:21:12 PM	Inj :	1
		Inj Volume :	1.000 µl

Acq. Method : C:\Chem32\1\Data\New 2025-06-12 14-20-20\DENEME.M
Last changed : 6/12/2025 2:20:20 PM by SYSTEM
Analysis Method : C:\Chem32\1\Data\New 2025-06-12 14-20-20\DENEME.M (Sequence Method)
Last changed : 6/24/2025 11:46:29 AM by SYSTEM

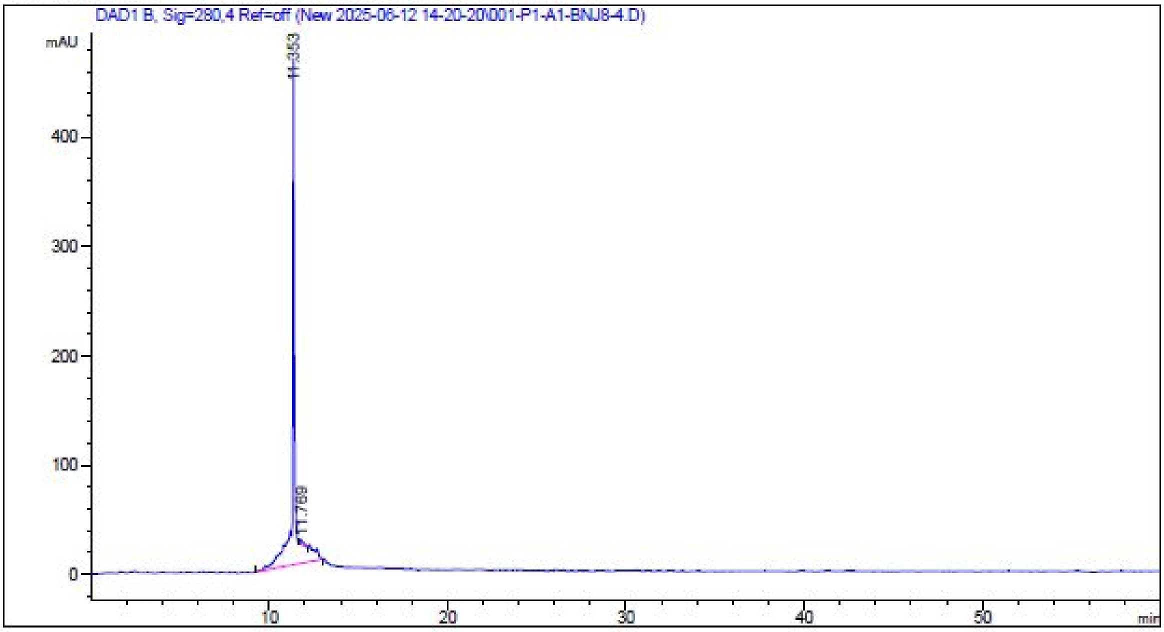

===
Area Percent Report
===

Sorted By : Signal
Multiplier : 1.0000
Dilution : 1.0000
Do not use Multiplier & Dilution Factor with ISTDs

Signal 1: DAD1 B, Sig=280,4 Ref=off

Peak #	RetTime [min]	Type	Width [min]	Area [mAU*s]	Height [mAU]	Area %
1	11.353	VV R	0.1449	5044.35400	463.72812	98.8354
2	11.769	VV T	0.1721	59.43682	4.55906	1.1646

Totals : 5103.79083 468.28718

Figure S21. HPLC chromatogram of compound **3h**

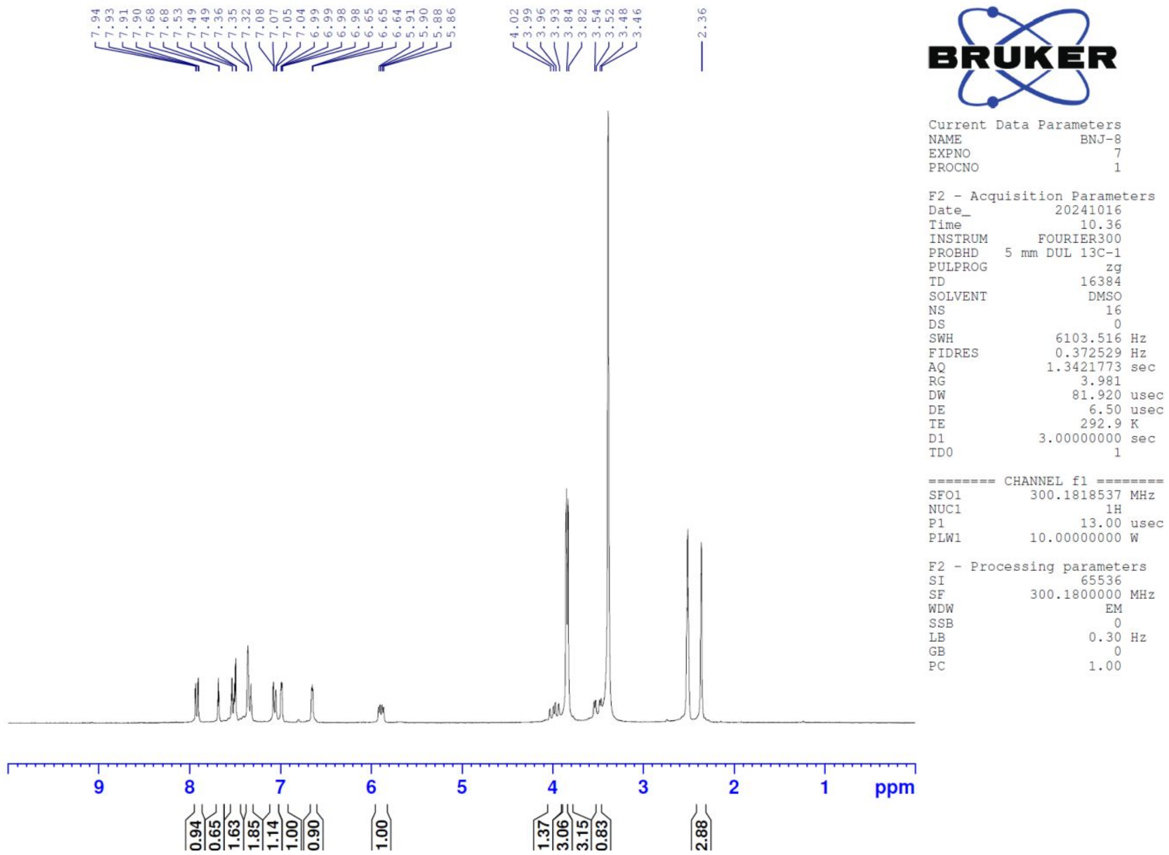

Figure S22. ^{1}H-NMR spectrum of compound **3h**

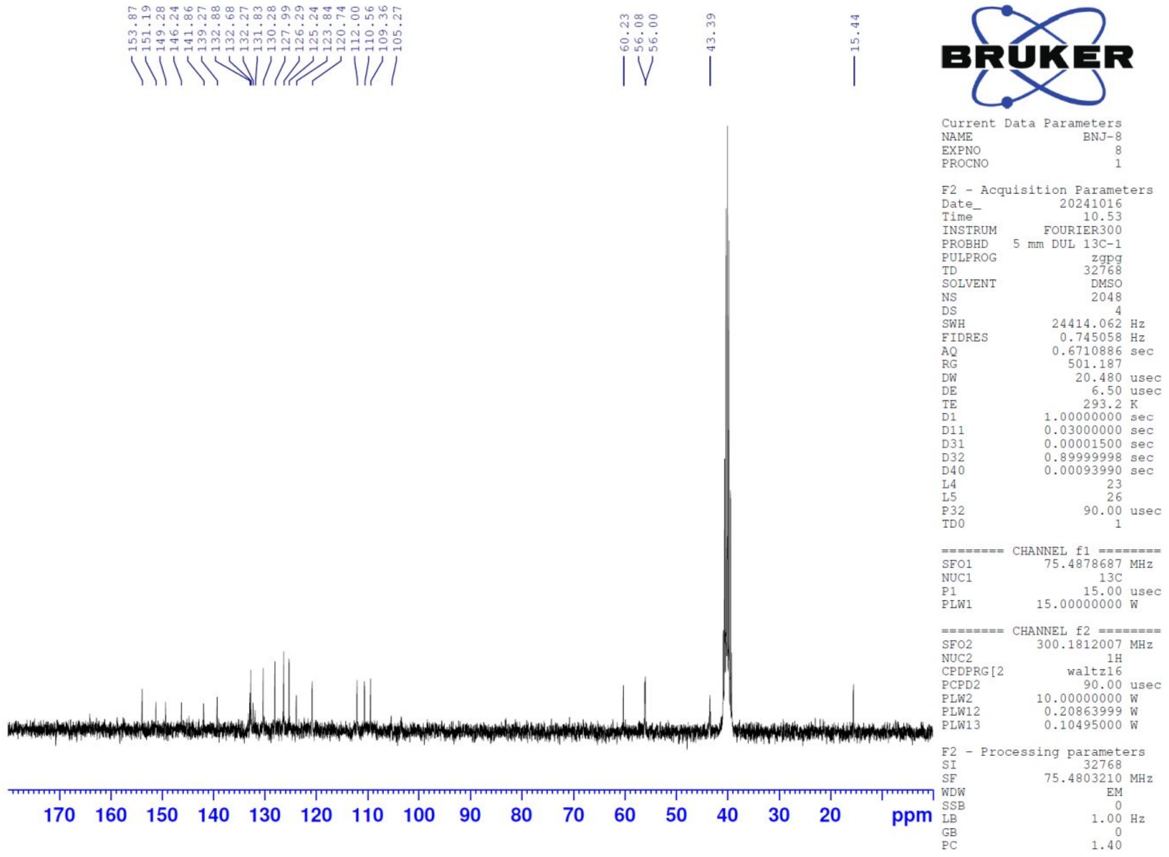

Figure S23. ^{13}C-NMR spectrum of compound **3h**

Data File C:\Chem32\1\Data\BNJ 2025-06-13 12-55-54\011-P1-A11-BNJ10.D
Sample Name: BNJ10

===

Acq. Operator : SYSTEM Seq. Line : 11
Acq. Instrument : HPLC DAD Location : P1-A11
Injection Date : 6/13/2025 4:25:36 PM Inj : 1
Inj Volume : 2.000 µl
Acq. Method : C:\Chem32\1\Data\BNJ 2025-06-13 12-55-54\DENEME.M
Last changed : 6/13/2025 12:55:54 PM by SYSTEM
Analysis Method : C:\Chem32\1\Data\BNJ 2025-06-13 12-55-54\DENEME.M (Sequence Method)
Last changed : 6/24/2025 11:43:16 AM by SYSTEM
(modified after loading) (Current integration events modified)

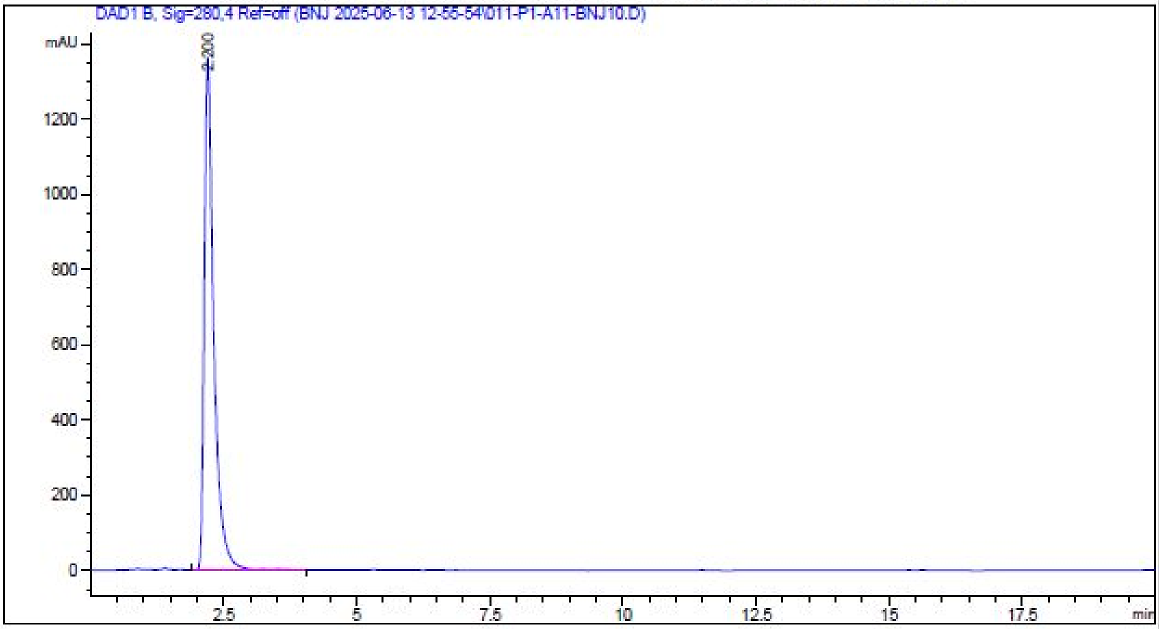

===

Area Percent Report

===

Sorted By : Signal
Multiplier : 1.0000
Dilution : 1.0000
Do not use Multiplier & Dilution Factor with ISTDs

Signal 1: DAD1 B, Sig=280,4 Ref=off

Peak #	RetTime [min]	Type	Width [min]	Area [mAU*s]	Height [mAU]	Area %
1	2.200	BV R	0.1813	1.69779e4	1363.40222	100.0000

Totals : 1.69779e4 1363.40222

Figure S24. HPLC chromatogram of compound **3i**

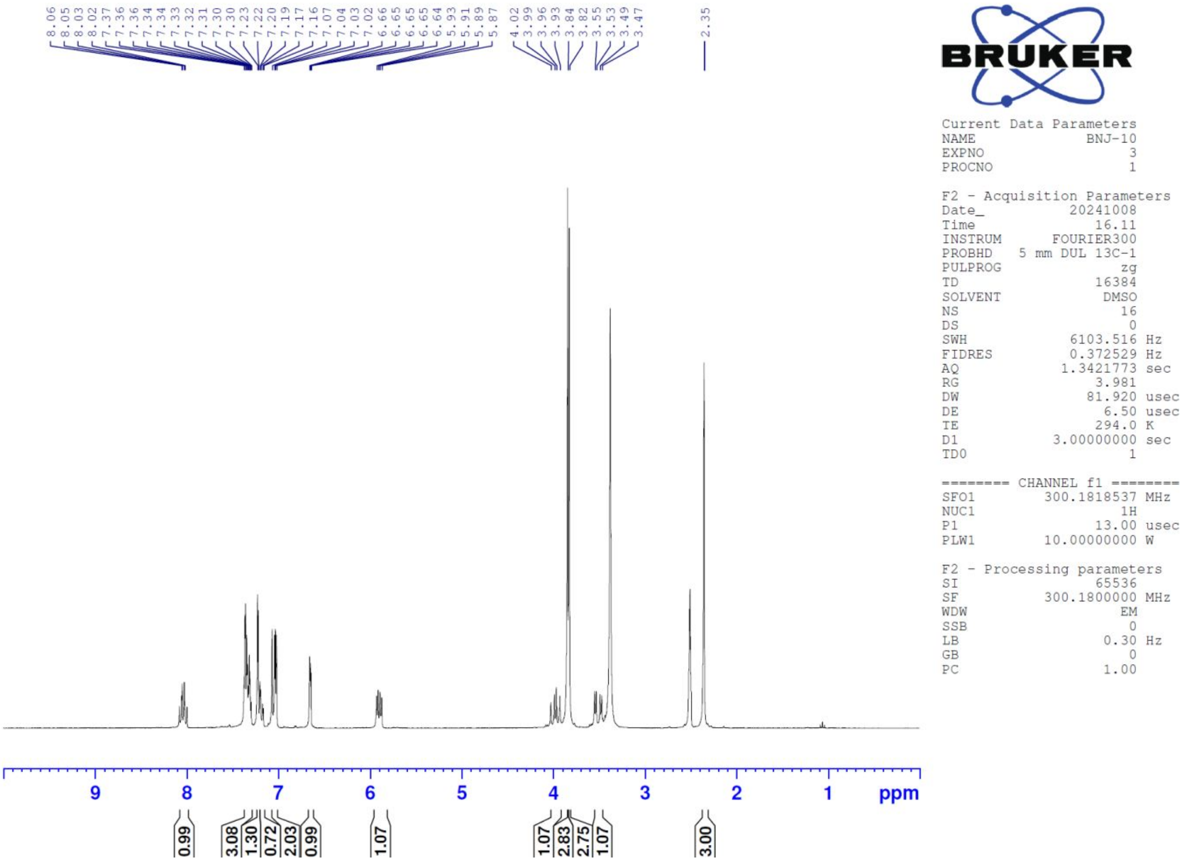

Figure S25. ^{1}H-NMR spectrum of compound **3i**

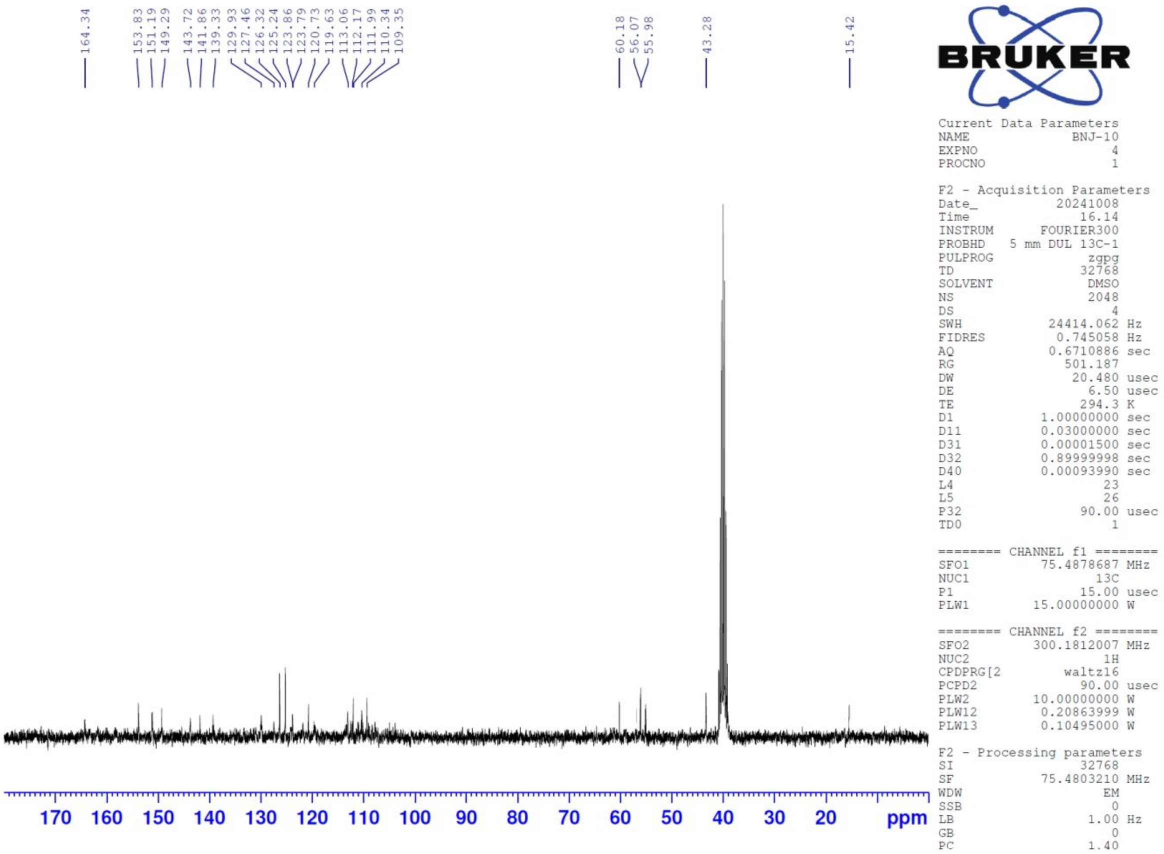

Figure S26. ^{13}C-NMR spectrum of compound **3i**

Data File C:\Chem32\1\Data\BNJ 2025-06-13 12-55-54\012-P1-B1-BNJ11.D
Sample Name: BNJ11

===

Acq. Operator : SYSTEM Seq. Line : 12
Acq. Instrument : HPLC DAD Location : P1-B1
Injection Date : 6/13/2025 4:46:31 PM Inj : 1
Inj Volume : 2.000 µl
Acq. Method : C:\Chem32\1\Data\BNJ 2025-06-13 12-55-54\DENEME.M
Last changed : 6/13/2025 12:55:54 PM by SYSTEM
Analysis Method : C:\Chem32\1\Data\BNJ 2025-06-13 12-55-54\DENEME.M (Sequence Method)
Last changed : 6/24/2025 11:43:34 AM by SYSTEM
(modified after loading) (Current integration events modified)

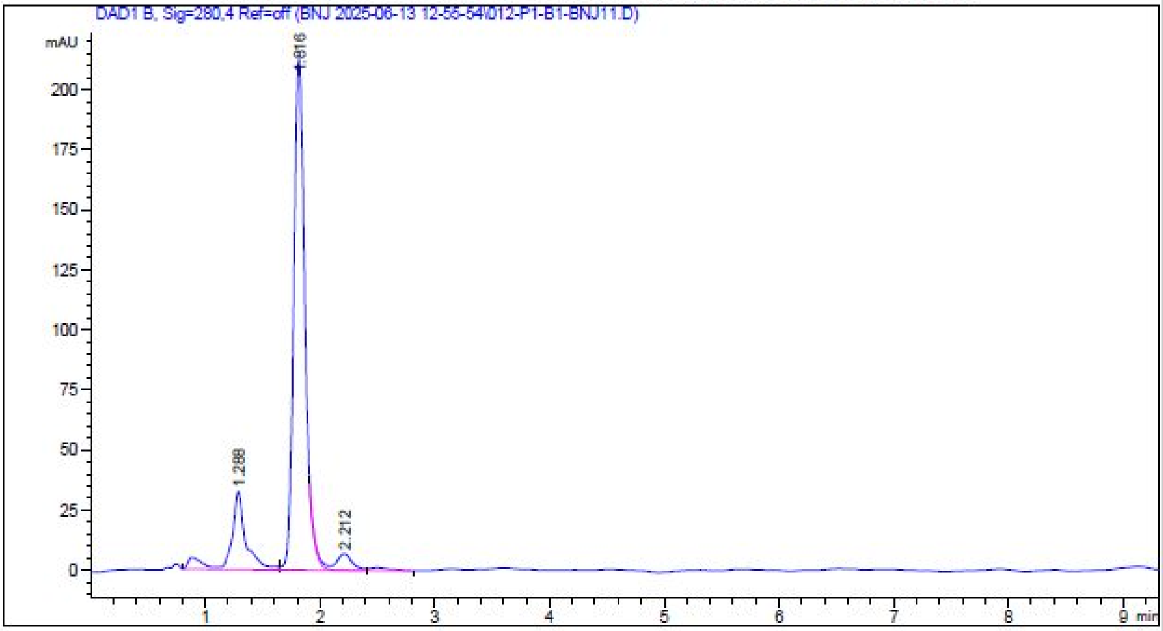

===

Area Percent Report

===

Sorted By : Signal
Multiplier : 1.0000
Dilution : 1.0000
Do not use Multiplier & Dilution Factor with ISTDs

Signal 1: DAD1 B, Sig=280,4 Ref=off

Peak #	RetTime [min]	Type	Width [min]	Area [mAU*s]	Height [mAU]	Area %
1	1.288	VV R	0.1277	304.51471	32.39231	16.4889
2	1.816	VV R	0.1043	1465.46741	213.02838	79.3523
3	2.212	VV E	0.1566	76.80421	6.97014	4.1588

Totals : 1846.78632 252.39083

Figure S27. HPLC chromatogram of compound **3j**

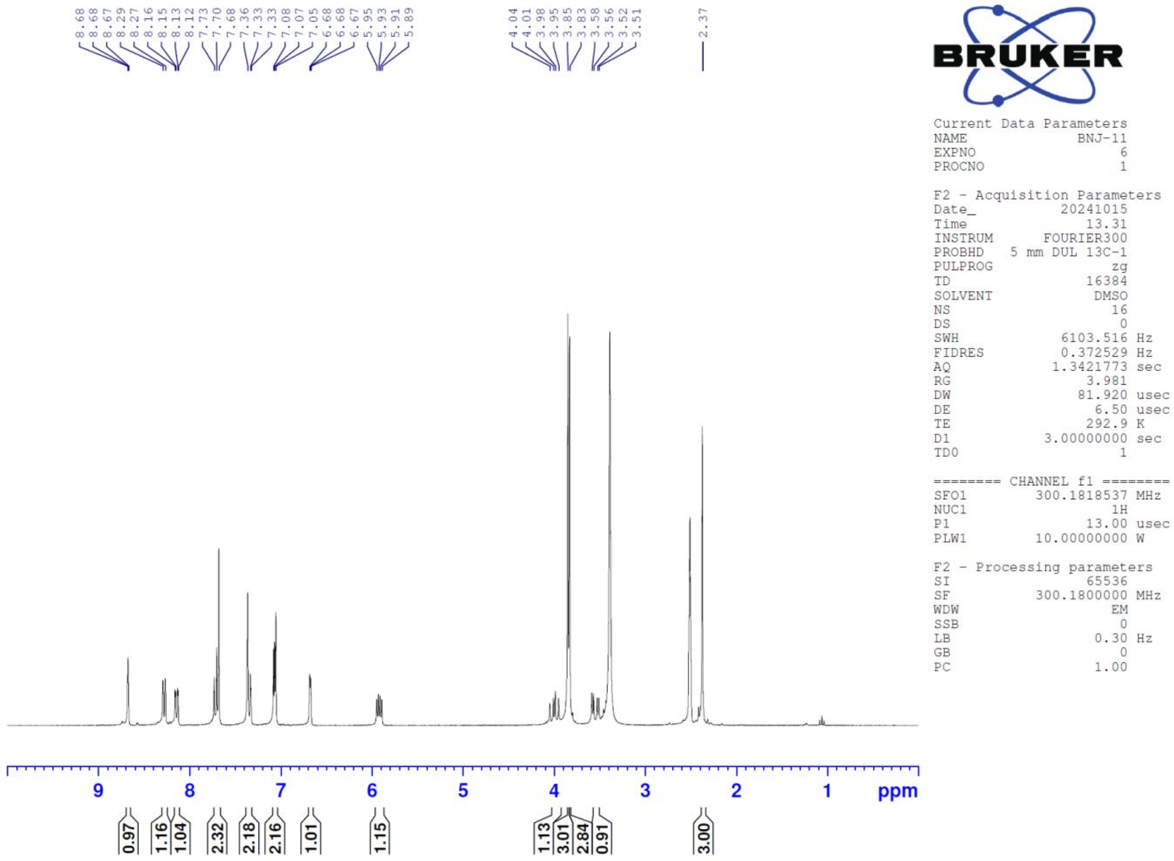

Figure S28. ^{1}H-NMR spectrum of compound **3j**

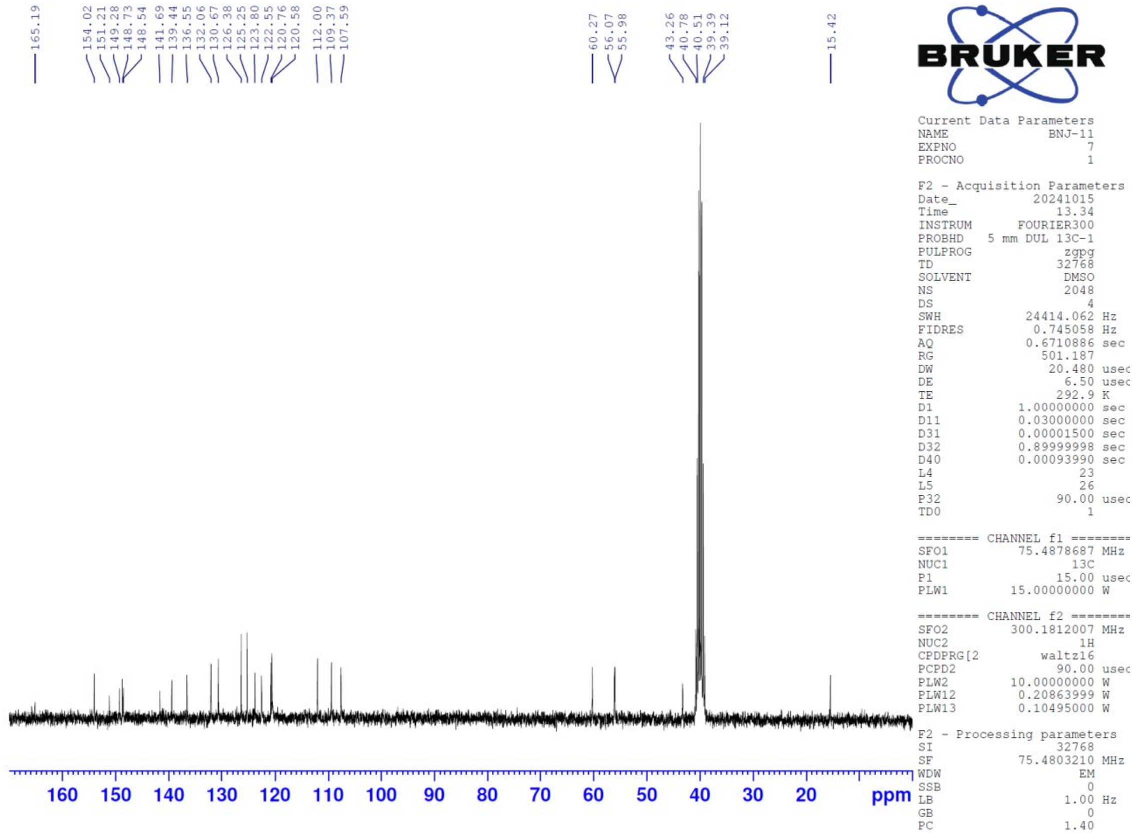

Figure S29. ^{13}C-NMR spectrum of compound **3j**
